# Highly Conductive PEDOT:PSS: Ag Nanowire-Based Nanofibers for Transparent Flexible Electronics

**DOI:** 10.1021/acsami.4c00682

**Published:** 2024-04-03

**Authors:** Xenofon Karagiorgis, Dhayalan Shakthivel, Gaurav Khandelwal, Rebecca Ginesi, Peter J. Skabara, Ravinder Dahiya

**Affiliations:** † James Watt School of Engineering, University of Glasgow, Glasgow G128QQ, U.K.; ‡ School of Chemistry, University of Glasgow, Glasgow G128QQ, U.K.; § Bendable Electronics and Sustainable Technologies (BEST) Group, Northeastern University, Boston, Massachusetts 02115, United States

**Keywords:** electrospinning, conductive fibers, transparent conductive electrodes, flexible electronics, silver nanowires, PEDOT:PSS

## Abstract

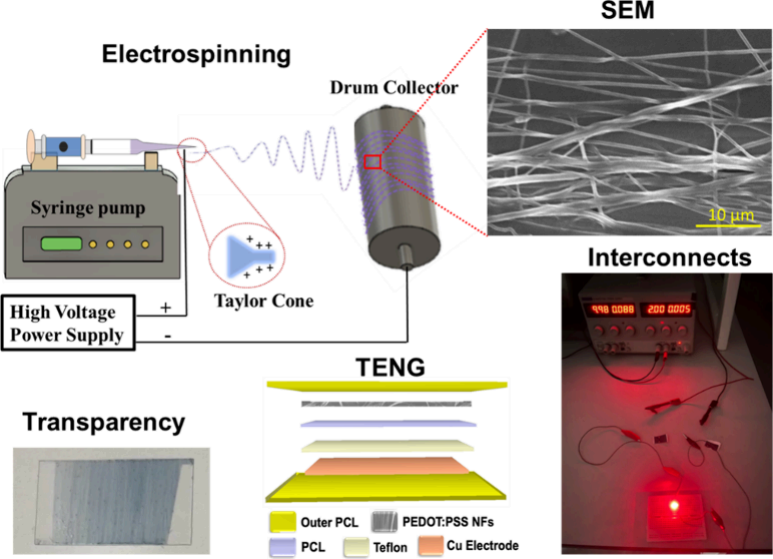

Highly conductive, transparent, and easily available materials are needed in a wide range of devices, such as sensors, solar cells, and touch screens, as alternatives to expensive and unsustainable materials such as indium tin oxide. Herein, electrospinning was employed to develop fibers of PEDOT:PSS/silver nanowire (AgNW) composites on various substrates, including poly(caprolactone) (PCL), cotton fabric, and Kapton. The influence of AgNWs, as well as the applied voltage of electrospinning on the conductivity of fibers, was thoroughly investigated. The developed fibers showed a sheet resistance of 7 Ω/sq, a conductivity of 354 S/cm, and a transmittance value of 77%, providing excellent optoelectrical properties. Further, the effect of bending on the fibers’ electrical properties was analyzed. The sheet resistance of fibers on the PCL substrate increased slightly from 7 to 8 Ω/sq, after 1000 bending cycles. Subsequently, as a proof of concept, the nanofibers were evaluated as electrode material in a triboelectric nanogenerator (TENG)-based energy harvester, and they were observed to enhance the performance of the TENG device (78.83 V and 7 μA output voltage and current, respectively), as compared to the same device using copper electrodes. These experiments highlight the untapped potential of conductive electrospun fibers for flexible and transparent electronics.

## 1 Introduction

Flexible and transparent conductive electrodes (TCE) are of interest in several applications such as touch interfaces,^1−3^ organic light emitting diodes (OLEDs),^4^ interactive displays,^5,6^ energy storage,^7−9^ and energy harvesting.^10,11^ Thus, far, indium tin oxide (ITO) has been the most used TCE material as it offers excellent optoelectronic properties (e.g., high transparency >85% and low sheet resistance (10–100 Ω/sq)).^12^ Despite such attractive attributes and, as a result, the commercial demand (>20% annual increment), it is challenging to rely on ITO because of high manufacturing cost and the scarcity of indium. Further, intrinsic properties such as brittleness, high temperature process requirement, and vacuum processing limit the use of ITO to electronics on conventional planar electronics. The higher temperatures possible on rigid and planar substrates (contributing to the attractive optoelectronic properties) are not achievable on plastic substrates due to the temperature limitations of the plastic substrates. Further, circuit patterning of ITO on flexible plastic films is expensive due to handling damages, which increase in probability as the circuit area increases. Emerging applications such as foldable displays and wearable health monitoring systems require flexible, lightweight, and durable electronics, which can reliably work despite extreme deformations such as bending and twisting.^13−15^ However, under bending conditions, ITO on a flexible substrate is prone to delamination and cracking.^16−19^ These challenges have motivated researchers to search for alternative TCE materials with figures of merits similar to ITO. These include (i) high transparency (>80%), (ii) low sheet resistance (<100 Ω/cm), (iii) high flexibility and stability under various mechanical deformations, (iv) large-area processability, (v) low cost, (vi) room temperature (RT) processing, and (vii) being easily available, i.e., abundant supply. In this regard, metals (nanoparticles or nanowires), carbon nanotubes (CNT), graphene and its derivatives, and conductive polymers have been explored. Conductive polymers are particularly attractive because of their inherent flexibility, tunable optoelectronic properties, simple synthesis, and low temperature for processing.^20,21^ Among these, the most common conductive polymer for transparent electrodes is poly(3,4-ethylene dioxythiophene):polystyrene (PEDOT:PSS), which offers high conductivity (ionic and electronic). Further, PEDOT:PSS is biocompatible, nontoxic, and low cost. The distinctive properties that allow PEDOT:PSS to stand out from other conducting polymers (such as doped poly(acetylene), poly(aniline), and poly(thiophene)) are its high electrical conductivity (∼100 S/cm) and high transparency (∼90%), which are comparable with conventional electrode materials.^22−24^

The techniques that are widely used to produce transparent electrodes include spin coating, screen printing, spray deposition, sputtering,^25^ and electrospinning. Notably, electrospinning has been extensively used over the last two decades to develop high aspect ratio nanofiber-based devices due to its suitability for large-area devices, low cost, coating uniformity, RT processing, alignment, tunability of fiber morphology, and dimensions in the sub-200 nm diameter range.^26^ Electrospinning of conducting polymers yields fibers with a high surface area, high permeability, flexibility, and excellent interconnectivity that allow them to be used as conductive electrodes. Moreover, compared to PEDOT:PSS thin films, a large-area, aligned fiber morphology possesses high flexibility along the longitudinal axis (i.e., spinning direction), which helps to retain the original electrical characteristics under multiple bending cycles. Despite their superior properties, electrospun conductive fibers have electrical properties that fall in the range of semiconductors (from 10^–6^ to 10^1^ S/cm)^27−32^ since they are usually incorporated with nonconductive electrospinnable polymers. Thus, efforts toward an improvement in the electrical properties of fibers have been made by applying deposition methods such as electroplating,^33^ dip coating,^34,35^ metal deposition,^36^ vapor phase polymerization,^37,38^ vapor exposure,^39^ oxidative polymerization,^40^ or in situ polymerization^41^ and other techniques.^42^ The postprocessing methods have negative consequences in terms of overall electrode performance, cost, and applicability.

To address this problem, in this work, we have applied a holistic single-step electrospinning process with a simple post-treatment process for highly conducting, flexible, and transparent fiber electrodes. The fibers were obtained by electrospinning of PEDOT:PSS mixed with silver nanowires (AgNWs). The effect of the AgNWs’ aspect ratio and filler concentration on the optoelectrical properties of the electrospun fibers was studied. After solution optimization, the effect of the electrospinning voltage was also investigated. The PEDOT:PSS fibers with 7% v/v of AgNWs (aspect ratio ∼330) electrospun at 17 kV exhibited the best electrical properties (∼7 Ω/sq and 354 S/cm) with a transmittance value of ∼77%. A further increase in the AgNW concentration resulted in increased resistance and reduced transmittance due to their disorientation inside the fibers and the morphology of the developed fibers. The AgNWs were replaced by copper nanowires (CuNWs) for a performance comparison. The CuNWs showed inferior optoelectrical properties compared to the AgNWs. The optimized PEDOT:PSS fibers electrospun on different substrates (polyimide film, PCL film, and cotton fabric) were demonstrated as interconnects in an LED circuit before and after 1000 bending cycles. In addition, the fibers were tested as electrodes in a triboelectric nanogenerator and showed superior performance over metal electrodes, suggesting the suitability of the fibers to replace metal electrodes in devices.

Table S1 provides a comparison between conductive fibers formed through continuous electrospinning with conductive polymers from previous literature and the conductive fibers developed in this study. Meanwhile, Table 1 outlines a comparison between this study and other flexible transparent electrodes detailed in the literature. From both tables, it is observed that the fibers reported in this work demonstrate comparable electrical properties with other TCEs and notably outperforms conductive polymer fibers, with significantly lower sheet resistance and higher electrical conductivities. The TCEs based on techniques like spin coating and dip coating involve multisteps introducing complexity to the process with a lack of control.^43,44^ Furthermore, the best results on fibrous-based TCEs use expensive metals (palladium) and multistep complex process like a combination of electrospinning, electroplating, and dip coating.^45,46^ Other fibrous TCEs use metals like copper, which are susceptible to issues of corrosion and oxidation, hence affecting their long-term durability.^45−47^ Thus, the effectiveness of TCEs is not exclusively determined by their properties. It also depends on factors such as the ease of fabrication, flexibility, robustness, and their ability to adhere to various substrates without the need for additional treatments. These factors collectively determine their usability for real-time applications. Efforts to enhance the long-term durability of TCEs while preserving their optoelectrical and adhesive properties can significantly improve the overall performance of transparent electronics. The fibers presented in this study serve as an outstanding example, offering a promising pathway for future research and advancements in this field.

**Table 1:** Comparative Table of the Current Work with Literature^a^

**method**	**materials**	**substrate**	**sheet resistance**[Ω/sq]	**conductivity**[S/cm]	***T* [%]**	**ref**
spin coating	PEDOT:PSS/AgNWs	PDMS	9.05	N/A	84	(48)
spin coating	PVK/Ag/PEDOT:PSS	PET	10	N/A	82	(49)
dip coating/plasma treatment	Ti_2_CT_*x*_	Al_2_O_3_	63	N/A	89	(43)
spin coating	PEDOT:PSS/CuNWs	PVA-coated CPI	135	705	85	(50)
spraying	Ti_3_C_2_T_*x*_/AgNWs/PEDOT:PSS	PET	30	N/A	81	(51)
RF magnetron sputtering	ITO	PET	1.16	N/A	82	(52)
spin coating/photopatterning	PEDOT:PSS/HDD	PET	N/A	627	86	(53)
spin coating	6 layers of PEDOT:PSS/Cu NPs	SiO_2_/Si	62	N/A	N/A	(54)
roll-to-roll coating	AgNWs/PEDOT:PSS	PET	20	N/A	95	(55)
spin coating	rGO/PEDOT:PSS	PDMS	170	2000	92	(44)
spin coating	AgNW-MXene@PEDOT:PSS	PET	17	N/A	97.6	(56)
electrospinning	Cu NFs	Glass	50	N/A	90	(45)
electrospinning/electroplating/ion-exchange	PAN/Cu/Ag	PDMS	0.225	N/A	N/A	(46)
	PAN/Cu/Pd		324	N/A	N/A	
electrospinning	PVA/CNT/Cu	N/A	39		81	(47)
electrospinning	PEDOT:PSS/AgNWs	PCL film	7 ± 2	354	77	our work

a PEO: poly(ethylene oxide), DMF: dimethylformamide, PVK: poly(*N*-vinylcarbazole), Al_2_O_3_: aluminum oxide, PET: poly(ethylene terephthalate), CPI: clear poly(imide), PDMS: poly(dimethylsilane), PVA: poly(vinyl alcohol), NPs: nanoparticles, HDD: hexa-2,4-diyne-1,6-diol, PAN: polyacrylonitrile, and Pd: palladium

## 2 Results and Discussion

**Figure 1:**
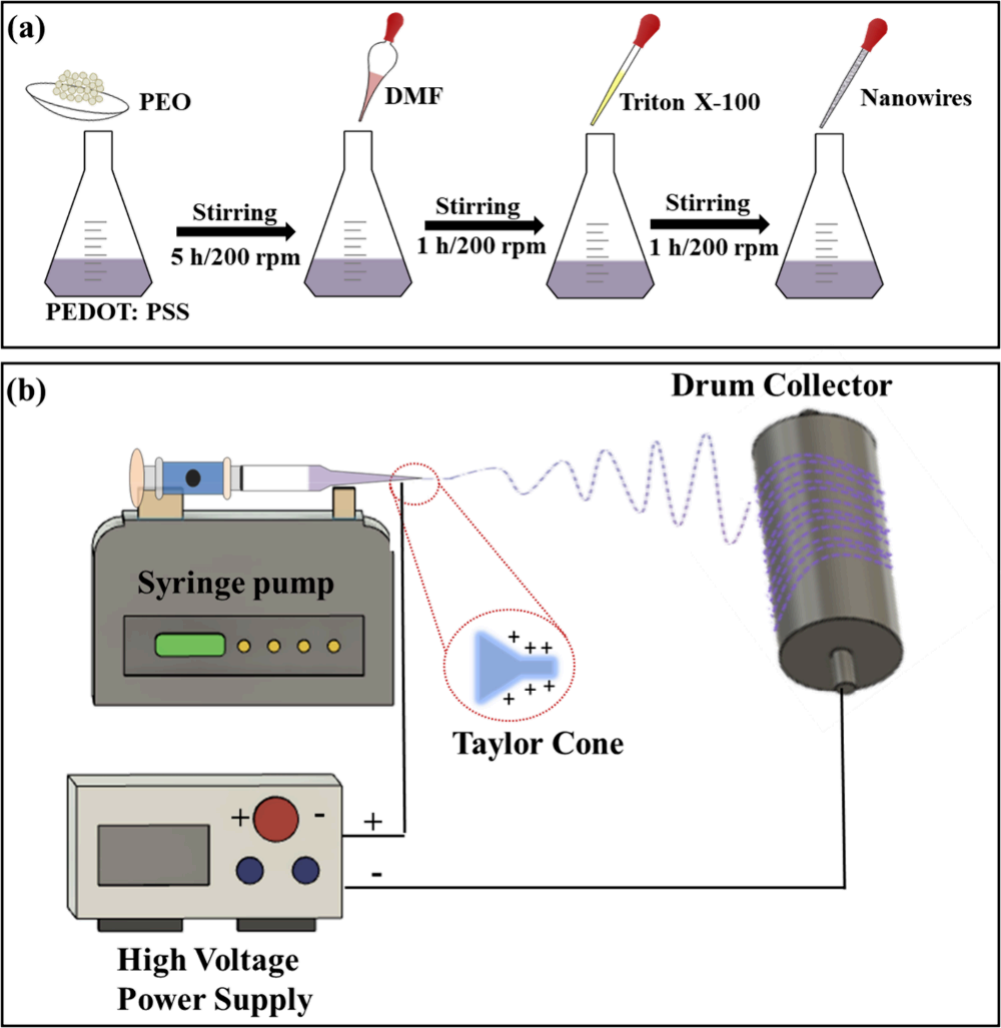
(a) Preparation of electrospinnable solutions and (b) schematic illustration of electrospinning.

Figure 1a depicts the method for the preparation of solutions containing conductive materials. Figure 1b shows a schematic representation of electrospinning. In PEDOT:PSS, PEDOT is in a doped state and is positively charged. The counteranions are part of the PSS component, which is an insulator, but it does not drastically affect the overall conductivity and the processed material, which reaches almost 100 S/cm. Applying an additive can improve the electrical conductivity significantly due to a conformational change of PEDOT. Therefore, DMF was used as an additive for the PEDOT:PSS solutions. The pure conductive polymers cannot be electrospun because their high conductivity hinders the formation of the Taylor cone. In addition, pure conductive polymers exhibit poor solubility and are quite brittle, causing mechanical instability. Hence, applying additives to formulations of electrospinnable polymers is mandatory to obtain good fibers of conductive polymers. Therefore, PVA and PEO were selected as the host polymers due to their excellent solubility in water. Tables S2 and S3 summarize the results on fiber morphology when different concentrations of PVA and PEO were added to PEDOT:PSS solutions for electrospinning. It was found that 2.25 wt % of PEO in PEDOT:PSS enables the formation of beadless fibers, while PVA cannot be used as the host polymer as no fibers were observed during the electrospinning process (Figure S1). Thus, PEO was selected as the ideal polymer matrix for the electrospinning of PEDOT:PSS solutions. To improve the conductivity of the PEDOT:PSS fibers, silver (Ag) and copper (Cu) nanowires were added to the solution.

**Figure 2:**
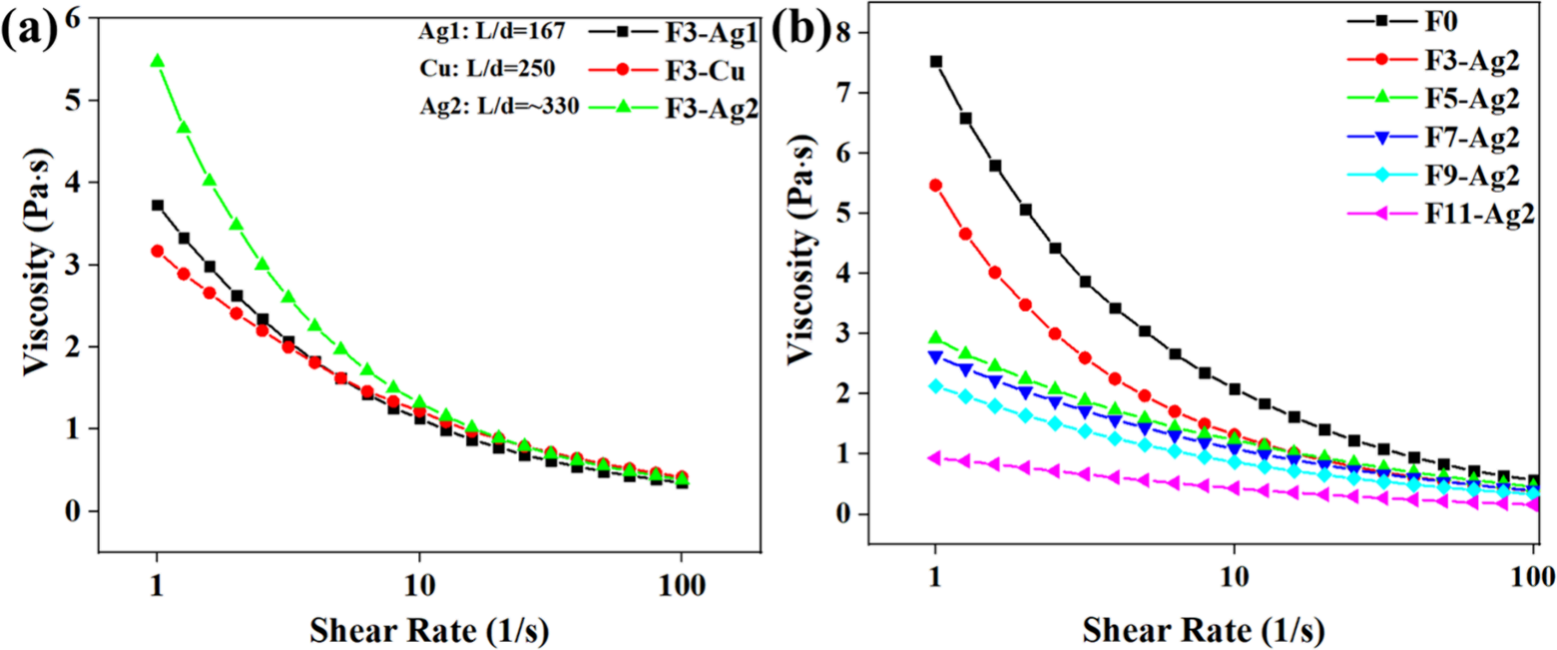
Dynamic viscosity measurements of (a) F3-Ag1 (3% v/v of Ag1 NWs), F3-Cu (3% v/v of Cu NWs), and F3-Ag2 (3% v/v of Ag2 NWs) and (b) F0, F3-Ag2 (3% v/v of Ag2 NWs), F5-Ag2 (5% v/v of Ag2 NWs), F7-Ag2 (7% v/v of Ag2 NWs), F9-Ag2 (9% v/v of Ag2 NWs), and F11-Ag2(11% v/v of Ag2 NWs).

The AgNWs with aspect ratios of 167 and 330 are named Ag1 and Ag2, respectively, and CuNWs are named Cu. In that regard, the samples were named as follows: F0 (0% v/v of nanowires), F3-Ag1 (3% v/v of Ag1 NWs), F3-Cu (3% v/v of CuNWs), F3-Ag2 (3% v/v of Ag2 NWs), F5-Ag2 (5% v/v of Ag2 NWs), F7-Ag2 (7% v/v of Ag2 NWs), F9-Ag2 (9% v/v of Ag2 NWs), and F11-Ag2 (11% v/v of Ag2 NWs). Figure 2a,b shows the effect of the nanowire concentration and aspect ratio on the viscosity of PEDOT:PSS solutions. The obtained results show a trend in viscosity with an increase in the nanowire concentration. From Figure 2a, it is clear that the size of nanowires affects the viscosity since the nanowires with a higher aspect ratio (F3-Ag2) formed a slightly more viscous (1.32 Pa·s) solution compared to samples F3-Ag1 (1.13 Pa·s) and F3-Cu (1.22 Pa·s) at a shear rate of 10 1/s. Figure 2b evaluates the viscosities of samples F0, F3-Ag2, F5-Ag2, F7-Ag2, F9-Ag2, and F11-Ag2. The addition of nanowires influences the viscosity of the solution. Since the nanowires are dispersed in IPA, higher loadings of nanowires include a larger amount of solvent. As a result, the viscosity of the samples decreased with the addition of larger quantities of nanowires. For instance, sample F7-Ag2 for 10/s has 1.09 Pa·s, which is considerably lower than the viscosity of F0 (without nanowires, 2.09 Pa·s) but higher than the viscosity of F11-Ag2 (0.435 Pa·s).

**Figure 3:**
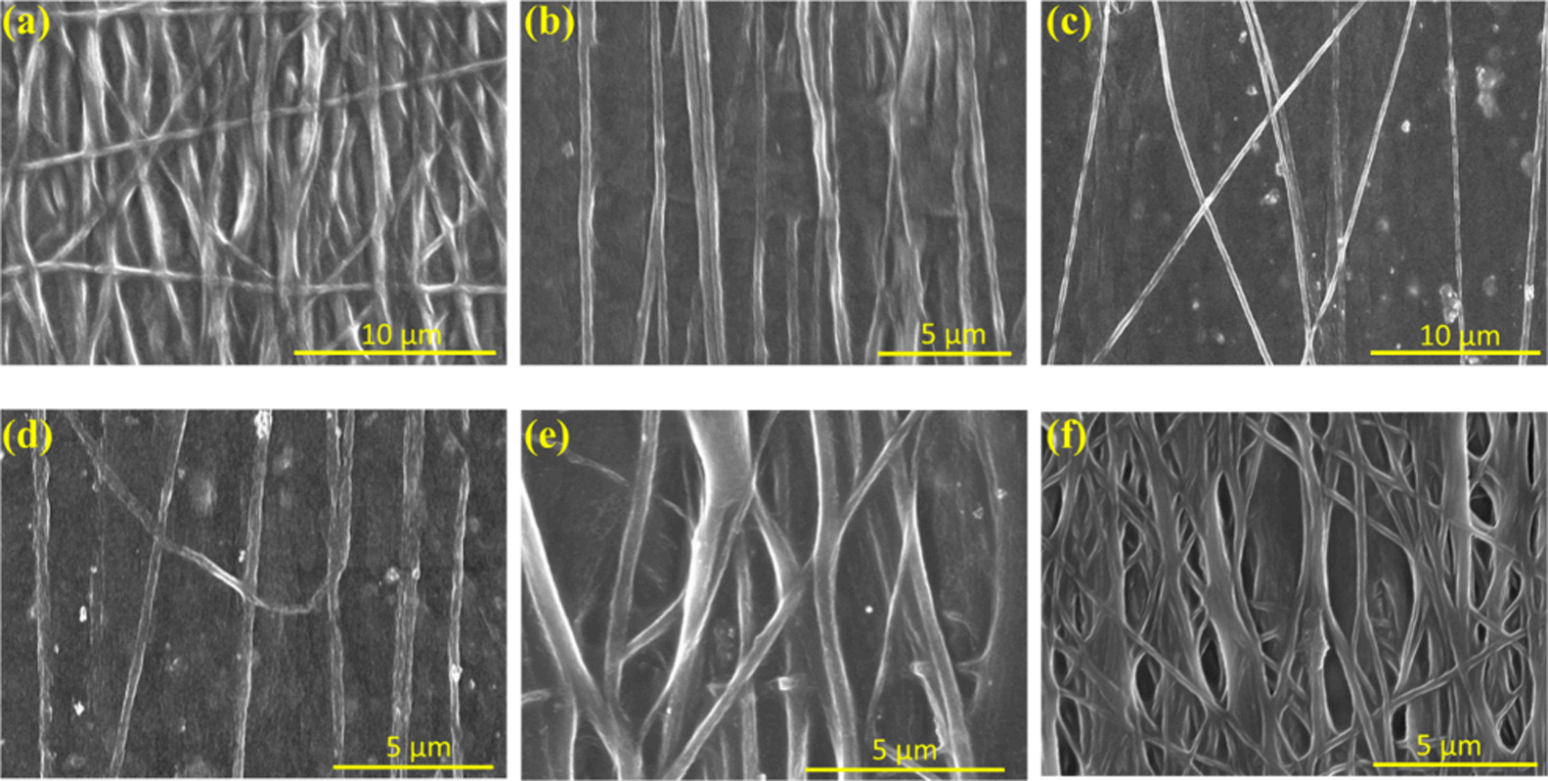
SEM images of the treated PEDOT:PSS fibers with different concentrations of Ag2: (a) F0, (b) F3-Ag2, (c) F5-Ag2, (d) F7-Ag2, (e) F9-Ag2, and (f) F11-Ag2.

After electrospinning, the fibers were immersed in ethylene glycol (EG) and annealed at 90 °C for 1 h to remove PEO from the fibers to further enhance the conductivity. Figure S2 depicts the morphology of the untreated fiber samples F0 and F3-Ag2 to F11-Ag2, and Figure S3 shows the structure of the untreated fibers F3-Ag1, F3-Cu, and F3-Ag2. Figure 3 shows the morphology of the fibers after the treatment. In both cases (treated and untreated), the electrospun fibers with both types of AgNWs (Figures S2a,c and S3 and Figure 3) appeared uniform and had a smooth surface. Moreover, it was observed that the fibers were long, continuous, and without beads, proving that the PEDOT:PSS-based solution with all the additives (dopants, surfactant, etc.), as well as the parameters that were used to obtain the electrospun fibers, was correct. However, the lower viscosity of solution F11-Ag2 and those with higher viscosity, such as samples F0 to F3-Cu, were challenging to electrospin. On the other hand, samples F3-Ag2 to F7-Ag2, with a viscosity of around 1 Pa·s, led to the formation of fibers with higher structural integrity. For the sake of comparison, all solutions were electrospun with the same parameters except the applied voltage during electrospinning, which was the minimum electrospinning voltage. However, in the case where PEDOT:PSS was mixed with CuNWs (F3-Cu), nonuniform fibers with beads were formed (Figure S2b), making them unsuitable for further device fabrication. Table S4 summarizes the average diameters of all the treated and untreated samples. The average diameters of the untreated samples F0 and F3-Ag2 to F11-Ag2 were 0.331, 0.63, 0.628, 0.459, 0.381, and 0.343 μm, respectively, while after the treatment, their average diameters were reduced to 0.316, 0.27, 0.323, 0.205, 0.243, and 0.218 μm confirming the removal of PEO after treatment. The EDX analysis (Figure S4) of sample F7-Ag2 shows the presence of Ag in the treated and untreated PEDOT:PSS fibers, while the presence of the elements oxygen (O), sulfur (S), and carbon (C) is from the PEDOT:PSS and PEO. It is noticeable that in both cases (treated and untreated fibers), the intensity for the Ag peaks remains the same, indicating that there is no influence of the treatment process on the AgNWs.

The effect of the applied voltage on the fiber morphology was investigated. The corresponding SEM images shown in Figure S5 reveal that the applied voltage (17, 19, and 21 kV) is an important parameter influencing the morphology and size of the fibers. PEDOT:PSS fibers obtained at an applied voltage of 17 kV (Figure S5a) were more uniform without beads and thinner compared to the fibers that were produced at applied voltages of 19 and 21 kV (Figure S5b,c). The average diameters of the fibers increase with an increase in applied voltage, i.e., 0.459 μm at 17 kV, 0.730 μm at 19 kV, and 0.856 μm at 21 kV.

**Figure 4:**
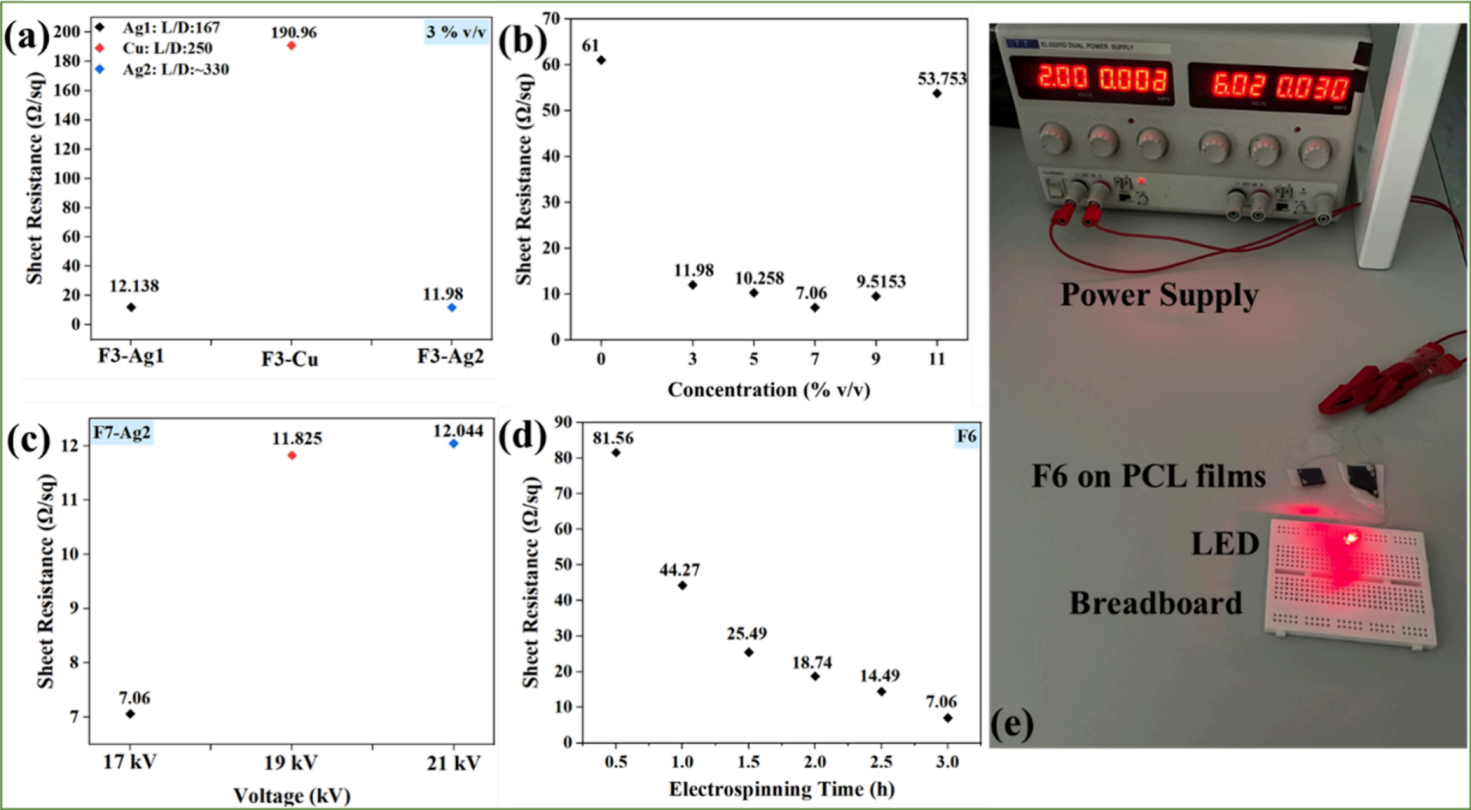
Sheet resistance of treated PEDOT:PSS fibers: (a) F3-Ag1, F3-Cu, and F3-Ag2 and (b) with Ag2 in various concentrations. (c) Sheet resistance of treated PEDOT:PSS fibers F7-Ag2 with different applied voltages, (d) sheet resistance of treated PEDOT:PSS fibers F7-Ag2 with varying electrospinning times, and (e) LED circuit with F7-Ag2 electrospun for 3 h.

The electrical properties of the fibers obtained from each solution were assessed using a four-point probe technique. To ensure the accuracy of these measurements, the fibers were directly placed onto PCL films. This was done to prevent any interference from the aluminum foil covering the collector during the electrospinning. Figure 4a evaluates the effect of different nanowires (3% v/v) on the electrical properties of the produced treated fibers. F3-Ag2 exhibited the lowest sheet resistance (11.98 Ω/sq) compared to F3-Ag1, whose sheet resistance was slightly higher (12.138 Ω/sq), and F3-Cu (190.96 Ω/sq). This may be due to the lower conductivity of copper (5.98 × 10^7^ S/m at 20 °C) compared to silver (6.30 × 10^7^ S/m at 20 °C). The low sheet resistance of the PEDOT:PSS fibers F3-Ag2 may be attributed to the aspect ratio of Ag2 (∼330), which is higher than the aspect ratios of Ag1 (167) and Cu (250). The longer nanowires extend continuous conductive pathways inside the fibers, improving the fibers’ electrical conductivity.^57^ Another reason may be attributed to the density of the fibers. In the case of F3-Ag1, fibers are less dense compared to the fibers on F3-Ag2, as illustrated in Figure S3a,c. Figure S6a shows the effect of the same nanowires on the untreated fibers. The untreated fibers exhibited high sheet resistance due to the presence of PEO. Moreover, to clearly prove the advantages of electrospinning over solution casting and spin coating, films were fabricated with the optimized solution of F3-Ag2. Films with the same post-treatment as the electrospun fibers exhibited significantly higher sheet resistance (1.02 kΩ/sq) than the fibers from the same solution. This confirms that the higher surface area of fibers and better interface lead to improved electrical properties. An additional factor contributing to the lower sheet resistance of the fibers over the films could be the end-to-end alignment of silver nanowires within the fibers, which provide a continuous path for the conduction of electrons.^58^ In the case of fibers with Cu, a sheet resistance of 32 kΩ/sq was measured prior to treatment. However, after treatment, a significant reduction to 190 Ω/sq was observed, which is considerably higher than the fibers with the different types of Ag. The morphology of the F3-Cu fibers (Figure S2b) may be one of the reasons why the conductivity is considerably lower than the other samples.

Next, the effect of the Ag2 concentration on the sheet resistance of PEDOT:PSS fibers was analyzed (Figure 4b). Fibers without nanowires (F0) are less conductive (61 Ω/sq) compared to the fibers F3-Ag2, F5-Ag2, F7-Ag2, F9-Ag2, and F11-Ag2, in which PEDOT:PSS was mixed with 3, 5, 7, 9, and 11% v/v of Ag2, respectively. Specifically, the sheet resistances for the samples were 11.98 Ω/sq (F3-Ag2), 10.258 Ω/sq (F5-Ag2), 7.06 Ω/sq (F7-Ag2), 9.51 Ω/sq (F9-Ag2), and 53.753 Ω/sq (F11-Ag2). Similar to the earlier observation, the sheet resistance of all the samples was higher before the post-treatment process (Figure S6b). One such example is sample F7-Ag2, in which the sheet resistance was 291 Ω/sq (untreated) and was reduced to 7.06 Ω/sq (treated). Figure S6c shows the sheet resistance of untreated fibers electrospun at different voltages. As well as the SEM analysis, the considerably reduced sheet resistance of the treated fibers confirms the removal of PEO after the post-treatment process. In addition, the lower sheet resistance of the PEDOT:PSS fibers with the nanowires may be attributed to the interfacial interaction between PEDOT:PSS and the AgNWs, which leads to a higher degree of organization of the polymer chains.^59^ The organic–inorganic interaction between the polymeric matrix and nanowires generates an interface that provides more electron pathways and works as a conductive bridge for carrier transport. Hence, the interconnectivity in the fibers is considerably improved, which enhances the electrical conductivity. However, it is noticeable that after F7-Ag2, the sheet resistance started to increase gradually, indicating that having more nanowires in the PEDOT:PSS solution impacts their alignment in the electrospun fibers, decreasing their conductivity.

The 7% v/v (F7-Ag2) concentration of AgNWs showed the lowest sheet resistance compared with the other formulations. The conductivity can be calculated from sheet resistance using the following equations:
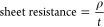
1where ρ is the resistivity and the thickness of the fibrous mat and σ is the conductivity of the fibrous mat:
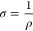
2

The thickness of the fibrous mat was obtained by using cross-sectional SEM imaging (Figure S7). The obtained thickness of the fibers (F7-Ag2) is around 1.94 μm. Following eqs 1 and 2, the conductivity (σ) of the sample F7-Ag2 was calculated to be approximately 354 S/cm. The thickness of the fiber mat can vary with the applied voltage and has a significant influence on the conductivity of the fibers. Therefore, the effect of applied voltage (17, 19, and 21 kV) on the fiber sheet resistance was evaluated. Figure 4c illustrates that the sheet resistance of the treated PEDOT:PSS fibers with 17 kV (7.06 Ω/sq) was considerably lower than the fibers, which were electrospun with applied voltages of 19 kV (11.825 Ω/sq) and 21 kV (12.044 Ω/sq). The poor electrical properties can be attributed to the fiber morphology and size as well as their density due to the increased applied voltage. Moreover, the sheet resistance of sample F7-Ag2 was also evaluated in different directions to the fiber alignment. Figure S6d illustrates that when the four-point probe was placed in the same direction as the treated fibers, their sheet resistance was slightly greater (7.06 Ω/sq) than when probes were placed perpendicular (10.98 Ω/sq) and diagonal (9.45 Ω/sq) to the fibers. However, these small variations in sheet resistance measured in different directions are still acceptable for the wide applicability of the electrode.

The influence of the electrospinning deposition time on the electrical properties of sample F7-Ag2 was investigated. Figure 4d demonstrates that when the fibers were electrospun on the PCL films for a longer time, their sheet resistance decreased steadily. The sample obtained from a 30 min deposition time gave a sheet resistance of 81.56 Ω/sq; the sheet resistance decreased to 7.06 Ω/sq when fibers were electrospun for 3 h due to increases in the density and effective thickness of the deposited fibers. Further increasing the electrospinning time resulted in a thicker fibrous mat with poor adhesivity.

The electrical measurement results confirm that the fiber sample F7-Ag2 electrospun for 3 h at an applied voltage of 17 kV shows the best results, and the sample was selected as the interconnect in a light emitting diode (LED), as shown in Figure 4e and Video S1 to demonstrate its performance in such a device. The examination suggests that the fibers can work as interconnects since the LED light turned on when 2 V was applied. For a proof of concept, sample F7-Ag2, electrospun for 30 min, was also tested as electrodes in an LED circuit, as demonstrated in Figure S8. The fibers are able to glow the LED but at a higher applied voltage of 3.35 due to higher sheet resistance than the ones electrospun for 3 h.

**Figure 5:**
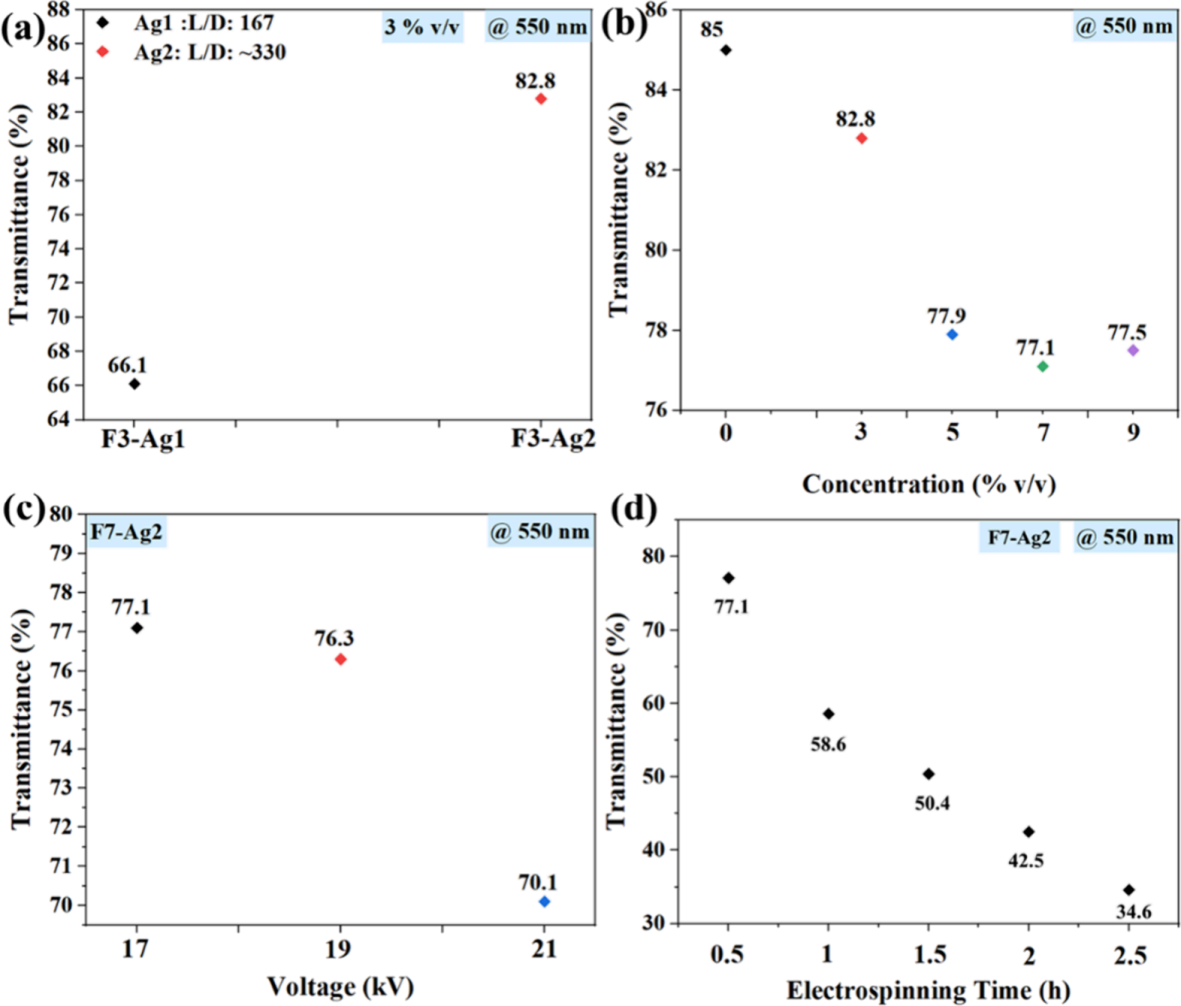
Transmittance of treated PEDOT:PSS fibers (a) with 3% v/v of Ag1 and Ag2, (b) with Ag2 in various concentrations, (c) treated fibers with 7% v/v of Ag2 (F7-Ag2) with different applied voltages, and (d) treated fibers with 7% v/v of Ag2 (F7-Ag2) produced over different electrospinning times with images for each deposition time.

To investigate the optical properties of the conductive fibers, all the above samples (F0, F3-Ag1, F3-Ag2, F5-Ag2, F7-Ag2, and F9-Ag2) of PEDOT:PSS/AgNWs were electrospun on glass substrates for 30 min with the post-treatment process mentioned earlier. Figure S9 compares the transmittance of untreated (67%T) and treated (77%T) samples F7-Ag2, showing that it was improved, after the post-treatment. Figure 5 shows the optical characterization at 550 nm for the treated fibers prepared by electrospinning. Figure 5a compares the transmittance of the PEDOT:PSS fibers with F3-Ag1 (66.1%) and F3-Ag2 (82.8%). The improvement of the transmittance may be attributed to the number of interfaces inside the fiber composites. Nanowires with a higher aspect ratio (L/D) diminish the number of junctions, thereby increasing the optical transmittance.^60−63^ Nanowires with smaller diameters absorb less incident light, leading to better transmittances.^57^Figure 5b shows the transmittances of PEDOT:PSS fibers with different concentrations of Ag2. The PEDOT:PSS fibers without nanowires (F0) exhibited higher optical transmittance (∼85%) than the fibers with nanowires. The addition of nanowires influences the transmittance of the fibers, and this can be explained by the reversible proportional relationship between %T and the concentration of nanowires.^64^ However, after a certain concentration of nanowires (≥5% v/v), the transmittance saturates at ∼77%, i.e., for samples F5-Ag2, F7-Ag2, and F9-Ag2. The outcomes were expected since the densely arranged nanowires inside the PEDOT:PSS matrix create shades in the fibers and thus the increase in their concentration reduces the transmittance of the fibers.^65^

Next, samples of F7-Ag2 electrospun at different applied voltages (17, 19, and 21 kV) were also tested for optical transmittance. Figure 5c shows the transmittance of the fibers at different applied voltages. The highest transmittance of 77.5% was measured for fibers produced at an applied voltage of 17 kV followed by 19 kV (76.3%) and 21 kV (70.1%). The decrease in the transmittance of the fibers can be attributed to the influence of applied voltage on the fiber’s morphology and fiber structure, in which, as shown in Figure S5, fibers electrospun at 19 and 21 kV formed beads on their surfaces. Moreover, a higher applied voltage forces out more flow of the solution from the tip, producing fibers with larger diameters.^66^ Thus, sample F7-Ag2 electrospun at 17 kV exhibited the best performance, with a good trade-off between sheet resistance and optical transparency.

Figure 5d compares the transmittance of fibers F7-Ag2 electrospun for different times on glass substrates at 550 nm. Figure 5d also shows photographs of the electrospun fibers processed over different deposition times. It is evident that the deposition time significantly affects the transmittance of the fibers since, for 30 min of electrospinning, the best logo is clearly seen compared to the fibers of 2.5 h of electrospinning.

## 3 Performance Evaluation

**Figure 6:**
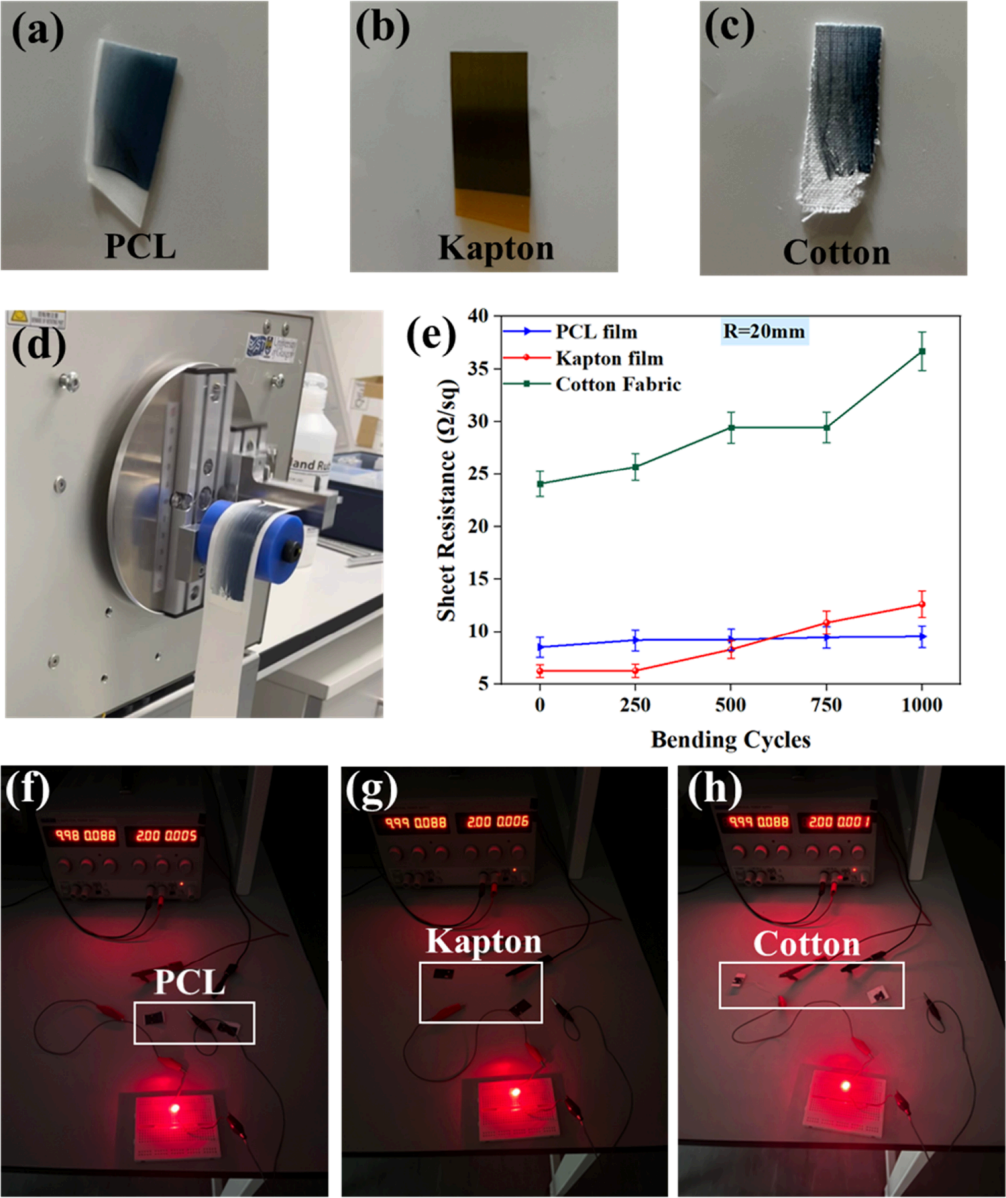
Treated PEDOT:PSS fibers (F7-Ag2) on the (a) PCL film, (b) Kapton film, and (c) cotton fabric. (d) Bending setup with cotton fabric/F7-Ag2. (e) Sheet resistance of PEDOT:PSS fibers on different substrates for different bending cycles. LED circuits with PEDOT:PSS fibers after 1000 bending cycles on the (f) PCL film, (g) Kapton film, and (h) cotton fabric.

Durable and long-lasting electrodes are desired for a wide variety of applications related to wearable systems and flexible devices. Herein, PEDOT:PSS fibers (F7-Ag2) were electrospun on substrates with different roughnesses (PCL, Kapton, and cotton fabric) for 3 h to examine the adhesivity as well as their performance after cyclic bending (Figure 6a–c). For all the considered substrates, it was observed that deposited fibers were strongly adhered to the substrate’s surface. To further examine the mechanical performance of the developed samples, bending tests on the samples were performed at a bending radius of 20 mm. The electrical properties of the fibers were monitored using the four-point probe method at regular intervals of 250 bending cycles over 1000 cycles. Figure 6e illustrates the average change of sheet resistance of the PEDOT:PSS fibers on different substrates for the 1000 bending cycles. It was observed that fibers on PCL films had negligible changes in their electrical properties, remaining at approximately 8 Ω/sq, whereas a noticeable increase was observed for the fibers on cotton fabric (from 24 to 36 Ω/sq) with some apparent cracks on them and 6–11.5 Ω/sq for the fibers on Kapton. The change in sheet resistance may be attributed to the different substrate roughnesses leading to a change in the adhesion on different substrates.

To further investigate the performance of the fibers after 1000 bending cycles as flexible conductive interconnects, they were connected to an LED circuit. This testing was to observe whether the fibers could turn on the LED lamp after the 1000 cyclic bending test. Figures 6f–h shows the LED circuit with the fibrous electrodes on PCL films, Kapton films, and cotton fabric, respectively. It is evident from the figures that despite the slight change in the electrical properties of the fibers, they were still able to glow LEDs at the same applied voltage of 2 V.

**Figure 7:**
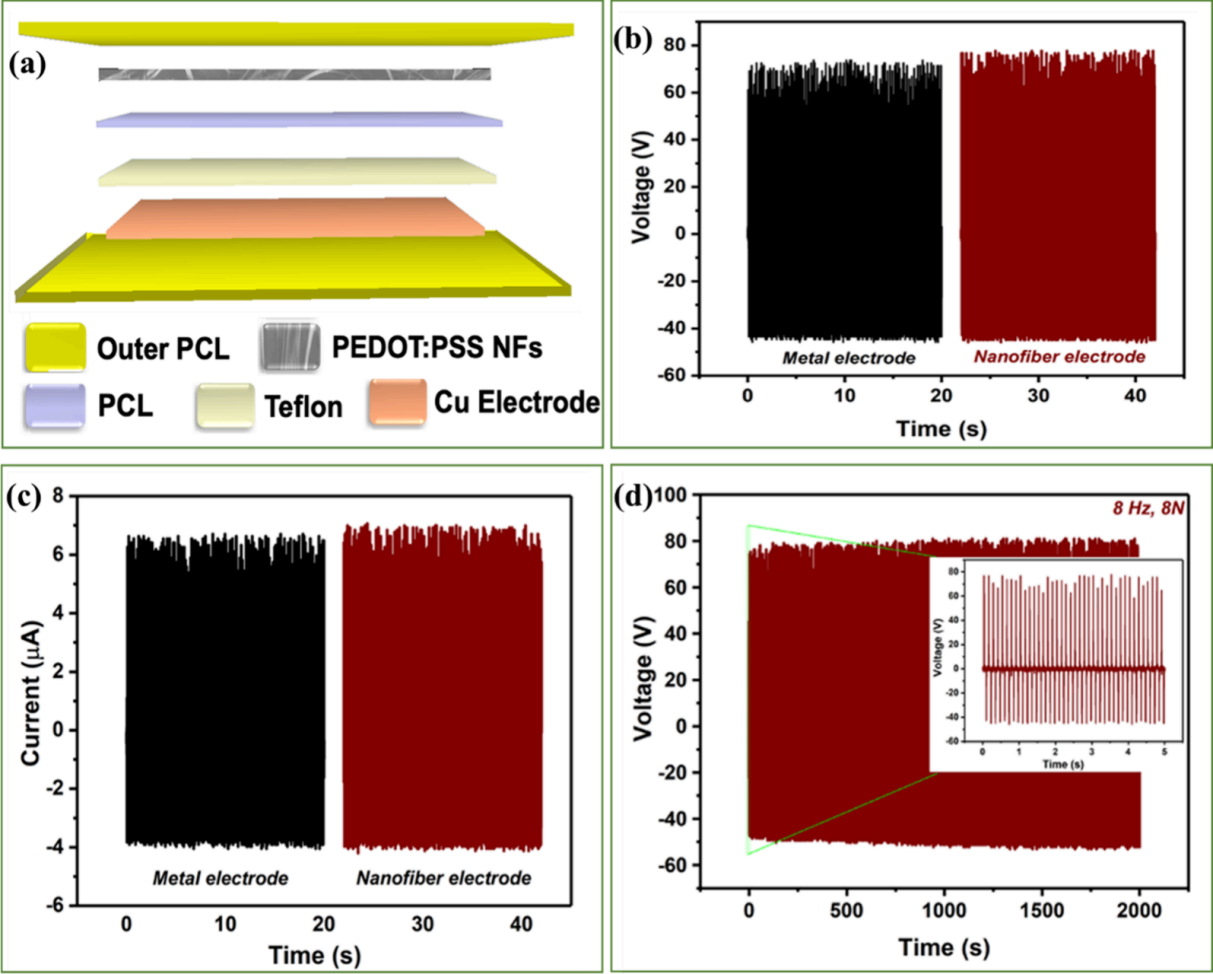
(a) 3D schematic (exploded view) of the triboelectric nanogenerator. (b) Voltage and (c) current profiles of the TENG with metal and fibers used as electrodes. (d) Stability of the fiber-based TENG.

The ability of PEDOT:PSS fibers (F7-Ag2) to work as electrodes has also been demonstrated by fabricating a triboelectric nanogenerator (TENG). The TENG is an energy-harvesting device that works through the coupling effect of triboelectrification (contact electrification) and electrostatic induction.^67,68^Figure 7a shows the schematic view of the fabricated vertical contact-separation TENG with PCL as the positive triboelectric layer, Teflon as the negative triboelectric layer, and copper as the electrode. For comparison, the copper electrode on the PCL active layer side was replaced with the electrospun PEDOT:PSS fibers to see the influence on the TENG’s performance. The metallic electrode-based TENG generated an output voltage of 73.9 V and an output current of 6.7 μA (Figure 7b,c). When the copper electrode was replaced with fibers, the electrical performance of the TENG was increased to 77.9 V and 7.1 μA. The increase in performance can be ascribed to a high surface area-to-volume ratio and better interface between the electrode and active layer leading to better charge transfer.^69^ The high surface area/volume ratio allows better charge collection from the electrode. Figure 7d shows the endurance test of the TENG device based on PEDOT:PSS NF for 16,000 cycles. The negligible or no output change confirms the stability of the fiber electrodes during continuous device operation. In addition, the output of the TENG was used to power the LEDs directly (Figure S10a) and also by using the fibers (F7-Ag2) as interconnects (Figure S10b and Video S1). The results obtained confirm the superior behavior of the PEDOT:PSS NFs, which will help in replacing expensive metallic transparent electrodes in energy harvesting, sensor, and energy storage devices and for other applications.

The polystyrene (PSS) part of PEDOT:PSS is hygroscopic in nature and can adsorb water in high-humid environments. The adsorption of water can lead to polymer swelling and has a negative impact on the conductivity. The change in resistance depends on the PEDOT to PSS ratio. The decrease in resistance was observed with an increase in humidity for the composition with a low PSS content. However, the sample with a high amount of PSS showed the opposite trend.^70,71^ Similarly, the output of the TENG decreases with the increase in humidity.^72^ The influence of the humidity depends on the hydrophobicity of the active layers. The polymers with high hydrophobicity like Teflon exhibit almost a negligible decrease in the output under high humidity conditions (70–80% RH). In the current work, the aim was to demonstrate the conductive nanofibers as electrodes for the TENG. In future studies, we will consider tuning the hydrophobicity of the TENG active layers to reduce the influence of humidity on the electrical performance of the device. Moreover, the focus will also be on the development of humidity-resistant highly conductive PEDOT:PSS-based electrospun fibers for flexible electronic devices.

## 4 Conclusions

Highly conductive and transparent electrospun PEDOT:PSS/AgNWs capable of operating as conductive paths in LED circuits and electrodes for triboelectric nanogenerators are presented. Electrospinning is an appealing fabrication technique due to its feasibility and potential applicability to large-area applications. The effect of different sizes of nanowires and concentration on the optoelectrical properties of the fibers was evaluated. The results suggest that solutions of PEDOT:PSS fibers with 7% v/v of Ag2 gave the best optoelectrical properties (354 S/cm, 77%T). The fibers were also electrospun on different substrates showing good adhesion and excellent electrical properties, even after subjecting them to 1000 bending cycles. Finally, a TENG with PEDOT:PSS/Ag2 fibers as electrodes exhibited comparable and even slightly higher voltage and current than the same TENG developed with metal electrodes.

## 5 Methods

### Materials

Poly(3,4-ethylenedioxythiophene):poly(styrenesulfonate) (PEDOT:PSS) (PH 1000) was purchased from Ossila, and poly(ethylene oxide) (PEO) (Mn: 100 kDa) was purchased from Alfa Aesar. Dimethylformamide (DMF), Triton X-100, AgNWs (0.5% isopropyl alcohol suspension in IPA) with aspect ratios of 120 (diameter × length, 50 nm × 6 μm) and 330 (diameter × length, 120–150 nm × 20 μm), the CuNWs (5 mg/mL in IPA, diameter × length, 100 nm × 25 μm, aspect ratio: 250), and poly(caprolactone) (PCL) (Mn: 45 kDa) were purchased from Sigma-Aldrich. Kapton substrates were purchased from DuPont, and cotton fabric was procured from a local seller. All chemical reagents were used as received without further purification.

### Solution Preparation

PEO (2.25 wt %) was added to a PEDOT:PSS solution and stirred for 5 h until completely dissolved. Then, 10% v/v of DMF was mixed and stirred for an hour to improve the conductivity of the processed PEDOT:PSS. Triton X-100 (2% v/v) was added to the doped solution as an effective liquid surfactant to change the surface tension of the solution and improve the wettability and uniformity. Finally, different nanowires (AgNWs and CuNWs) were added in different concentrations for comparison. The solutions were stirred for another hour for a uniform dispersion of nanowires. Solutions were prepared at room temperature and 200 rpm.

### Electrospinning

All electrospinnable solutions were inserted into a 20 mL syringe in the electrospinning setup (TL-PRO, TONGLI TL, Nanshan, Shenzhen, China). Experiments were carried out at room temperature and 30 ± 3% relative humidity. A stainless-steel needle (19 G) was arranged on the spinneret and connected with a positive voltage, 10 cm from the collector. A stainless-steel rotating drum (diameter = 30 cm, length = 10 cm) was covered with aluminum foil, connected with a negative voltage of 0.80 kV, and rotated at 1000 rpm. The flow rate was 1 mL/h. The formation of the fibers is shown in Video S1. Rectangular glass substrates (60 × 20 mm) and PCL films (5 cm × 5 cm) were placed on the aluminum foil for the optical and electrical characterization and attached with Kapton tape, respectively. The glass substrates were cleaned with isopropanol prior to electrospinning. Table S5 demonstrates the applied voltage for all PEDOT:PSS/nanowire solutions, and Figure 1b illustrates the electrospinning setup. Voltage was altered in each case because the viscosity of each solution was different in regard to the nanowires’ aspect ratios and concentrations. The voltages that have been used were the minimum electrospinning voltages (MEVs) in which uniform and beadless fibers were formed. After electrospinning, the fibers were washed in ethylene glycol for 10 min and then were calcinated for 3 h at 90 °C to remove PEO.

### Characterization

Dynamic viscosity measurements were performed with an Anton Paar Physica MCR101 rheometer. A cone-and-plate measuring system was used, with a 75 mm cone (angle = 1.000°) and a plate gap of 0.1 mm. Solutions (2 mL) were poured onto the plate for the measurement. The viscosity was measured under rotation shear rates in the range of 1–100 s^–1^. The temperature was maintained at 25 °C during all measurements using a water bath. Scanning electron microscopy (SEM) and energy-dispersive X-ray (EDX) analyses (SU8240, Hitachi) with accelerating voltages of 10 and 15 kV, respectively, were used to evaluate the morphology and thickness of the fibers and identify their elemental composition. The sheet resistance of the electrospun fibers was measured with four-point probe equipment (Ossila, Ltd.). The probes were positioned in line with even spacing (0.5 cm). Electrical current was passed through two outer probes (1 and 4), and the sheet resistance was obtained by measuring the change in the voltage. Transmittance measurements were conducted on a UV-2600 spectrophotometer (Shimadzu Ltd.), using rectangular glass substrates (22 × 40 mm, Menzel-Gläser). For bending tests, a Yuasa bending endurance setup (Yuasa System Co., Tokyo, Japan) was used.

## Supporting Information Available

Formation and deposition of the fibers on the collector and the use of the conductive fibers as interconnects and as the electrode for the TENG and interconnects to power LEDs (MP4)
A comparative table of electrospun nanofibers based on conductive polymers, optoelectrical characterization of the electrospun PEDOT:PSS-based fibers, and performance evaluation of the conductive fibers as interconnects and electrodes (PDF)
