# Supplementary material for: Highly Conductive PEDOT:PSS: Ag Nanowire-Based Nanofibers for Transparent Flexible Electronics
Source: ACS Appl Mater Interfaces. 2024 Apr 3;16(15):19551–62. doi: 10.1021/acsami.4c00682 (PMC11040574; doi:10.1021/acsami.4c00682)
Supplement: Supplementary file 2 — am4c00682_si_002.pdf [file am4c00682_si_002.pdf]

Highly conductive PEDOT:PSS - Ag Nanowires based nanofibres for transparent flexible electronics

Xenofon Karagiorgis[1,2], Dhayalan Shakthivel[3], Gaurav Khandelwal[1], Rebecca Ginesi[2], Peter J. Skabara[2] and Ravinder Dahiya[3*]

[1]James Watt School of Engineering, University of Glasgow, Glasgow, G128QQ, UK
[2]School of Chemistry, University of Glasgow, Glasgow, G128QQ, UK
[3]Bendable Electronics and Sustainable Technologies (BEST) Group, Northeastern University, Boston, MA 02115, USA

Corresponding author: r.dahiya@northeastern.edu

Supporting Information

Table S1: Comparative table

Conductive Polymer	Additives	Resistance [KΩ]/Sheet Resistance [Ω/sq]/ Resistivity [Ω.m]	Conductivity [S/cm]	Transmittance [%]	References
PEDOT:PSS	Chitosan/DMSO		7.63×10^{-5}	-	[1]
	PVA/DMSO		2×10^{-5}	-	[2]
	PVA		1.2×10^{-6}		[3]
	PVA/EG/Triton X-100		1.8	-	[4]
	PVA/DMSO		1.7×10^{-5}	-	[5]
	PVA/rGO		1.1×10^{-4}	-	[6]
	PVA/rGO		0.017		[7]
	PVA/ rGO		0.02		[8]
	PVA/boron carbon nitrogen nanotubes (BCNNTs)		2.36×10^{-5}		[9]
	PVA/AgNWs/DMSO	600 kΩ/sq	-	-	[10]
	PEO/DMF	12 kΩ		60	[11]
	PEO/Triton X-100/DMF		1.8	97	[12]

	PEO/CNF		13	-	13
	PEO/ PEG–PPG–PEG/ DMF	700 Ω/sq			14
	PEO		35.5	-	15
	PEO		13.82×10^{-3}	-	16
	PVP/DMF		2.34×10^{-12}	-	17
	PVP	10^{5} Ω· m		-	18
	PVP/ethyl alcohol/EG/1-ethyl-3-methylimidazole acetate		1.27×10^{-4}	-	19
	PVP/DMSO		1.6×10^{-5}	-	20
	PNN		11.2	-	21
	PNN/DMSO		29.4	-	22
	PEO/CSA		9.8×10^{-6}	-	23
	PEO/CSA	3.88 kΩ		-	24
	PEO/HCSA/MWCNT		48	-	25
	PEO/CSA		4.7	-	26
	PEO/CSA		5×10^{-4}	-	27
	PEO/CSA/Treated luffa (TL)		1.225×10^{-6}	-	28
	PU		4.577×10^{-3}	-	29
	PCL/HCSA		7.0×10^{-4}	-	30
**PANi**	PCL/PLGA/CSA		0.32×10^{-4}	-	31
	PAN/Ionic Liquid		2.62×10^{-6}	-	32
	PAN/CSA/Graphene		0.58	-	33
	PAN/CSA		3.415×10^{-5}	-	34
	PAN/DMF/Nickel NPs		0.047	-	35
	HCL-H_2SO_4/APS		52.9	-	36
	PVDF/MWCNT		2.75×10^{-9}	-	37
	PDLA/CSA		43.7×10^{-3}	-	38
	PLCL		1.445×10^{-4}	-	39
	S-HAP-PLA		0.05	-	40

	Chitosan/NaOH		2.6×10^{-7}	-	[41]
	PVB/DMF		18×10^{-6}	-	[42]
	Silk fibroin/CSA		0.5	-	[43]
	PDA/DMSO		1.31	-	[44]
	PVA/HCSA		3.6×10^{-5}	-	[45]
	PES/CSA		3.7×10^{-5}	-	[46]
	Gelatin/CSA		0.04	-	[47]
**PPy**	PCL/PG/HFP		3.7×10^{-4}	-	[48]
	PVCN/DBSA		0.3	-	[49]
	DBSA/PVA		1×10^{-10}		[50]
	PCL		1.549×10^{-4}	-	[51]
	AQSA/PVP		5.22×10^{-1}	-	[52]
	PEO/CS/Collagen		1.64	-	[53]
	PEO		1.44×10^{-3}	-	[54]
	PBAT		3.62×10^{-5}	-	[55]
	PELA		6.9×10^{-5}	-	[56]
**PTh**	PMMA-g-PTh/gelatin		1.6×10^{-3}	-	[57]
	S-PTh/gelatin		0.02×10^{-3}	-	[58]
	PCL		5.98×10^{-6}		[59]
	PEG_{2000}-b-$(PTh)_4$/PCL		1×10^{-3}	-	[60]
	PEG_{6000}-b-$(PTh)_4$/PCL		8×10^{-4}		[61]
	G_4-PTh/PCL		7×10^{-3}	-	[62]
	PMMA	40 Ω			[63]
	S-PCL–PTh		0.22		[64]
**PEDOT:PSS**	**PEO/ DMF/ Triton X-100/AgNWs**	**~7 Ω/sq**	**354**	**77**	**Our Work**

Table S2: PVA concentration and observation of electrospun fibres

**PVA wt%**<br><br>**Mw: 98KDa**	**Observations**	**Dopant**

**1**	Drops/no fibres	10 % v/v DMSO
**2**		
**3**		
**4**		
**5**		
**6**		
**10**		

Table S3: PEO concentration and observation of electrospun fibres

PEO %wt<br><br>Mn: 100 kDa	Observations	Dopant
**1**	Drops/no fibres	13 % v/v DMF
**2**	Fibres with beads	
**2.25**	Uniform fibres	

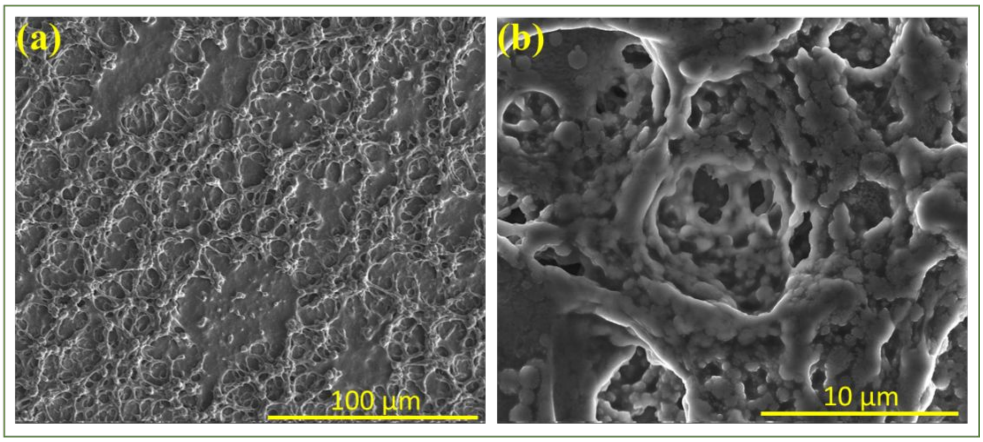

Figure S1. *SEM images of PEDOT:PSS/ 6 wt% PVA drops/no fibres at magnification of (a) 100 µm and (b) 10 µm.*

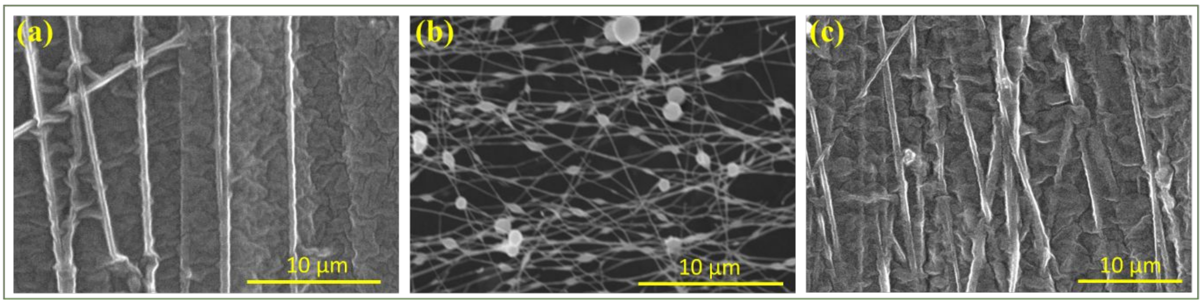

Figure S2. *SEM images of the untreated PEDOT:PSS fibres (a) F3-Ag1, (b) F3-Cu and (c) F3-Ag2.*

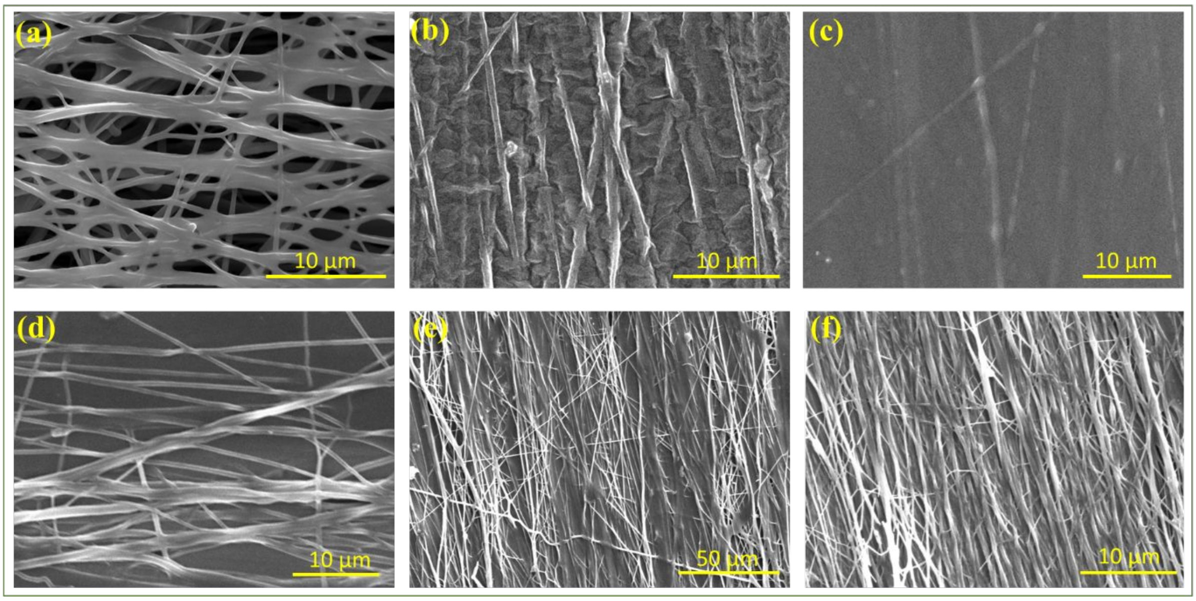

Figure S3. *SEM images of the untreated PEDOT:PSS fibres with different concentrations of Ag2 (a) F0, (b) F3-Ag2 (c) F5-Ag2 (d) F7-Ag2 (e) F9-Ag2 (f) F11-Ag2.*

Table S5: Average diameters of the untreated and treated samples.

**Sample**	**Untreated fibres**	**Treated fibres**
	**Average diameter [µm]**	**Average diameter [µm]**
**F0**	0.331	0.316
**F3-Ag1**	0.205	-
**F3-Cu**	0.442	-
**F3-Ag2**	0.630	0.270
**F5-Ag2**	0.628	0.323
**F7-Ag2**	0.459	0.205
**F9-Ag2**	0.381	0.243
**F11-Ag2**	0.343	0.218

**F7-Ag2 (19 kV)**	0.730	-
**F7-Ag2 (21 kV)**	0.856	-

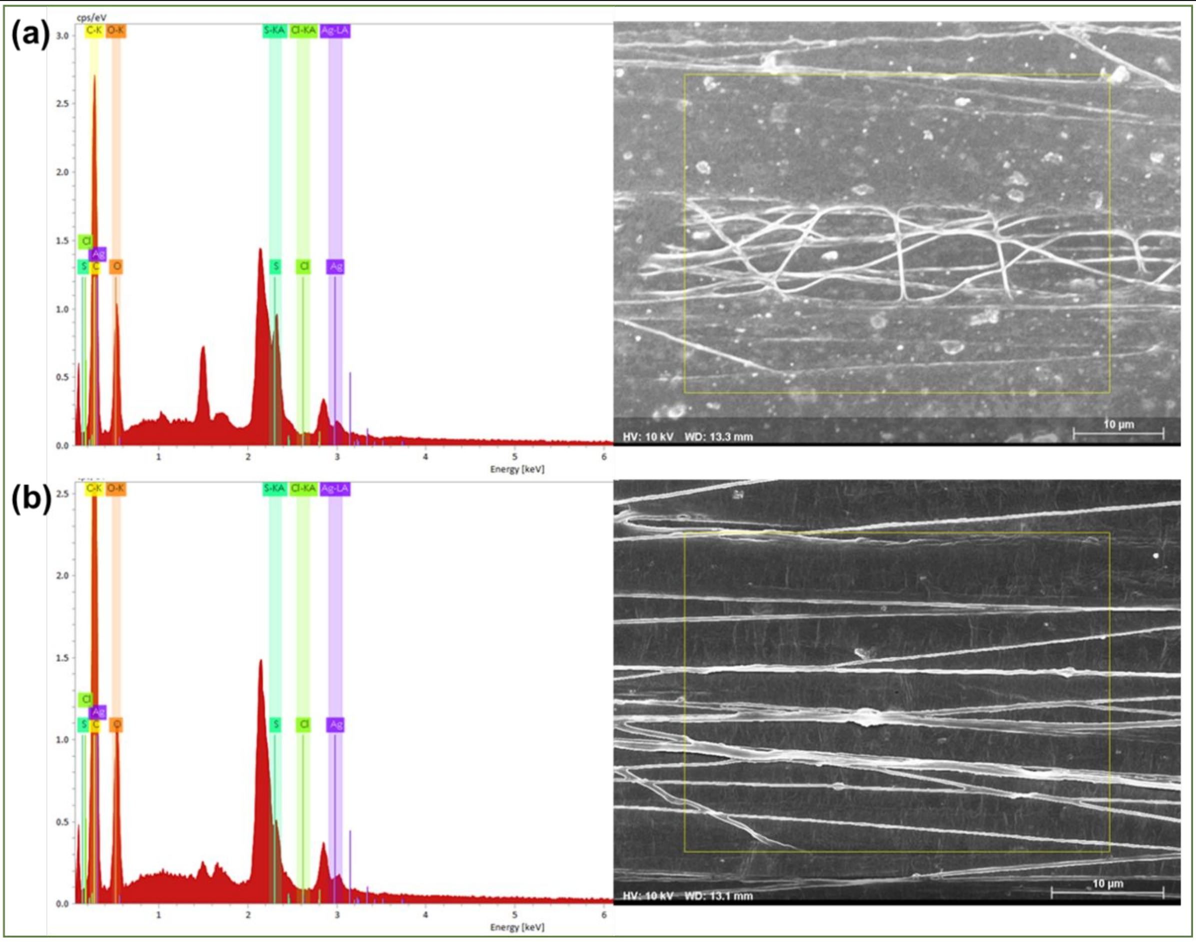

Figure S4. *EDX analysis of (a) treated and (b) untreated F7-Ag2*

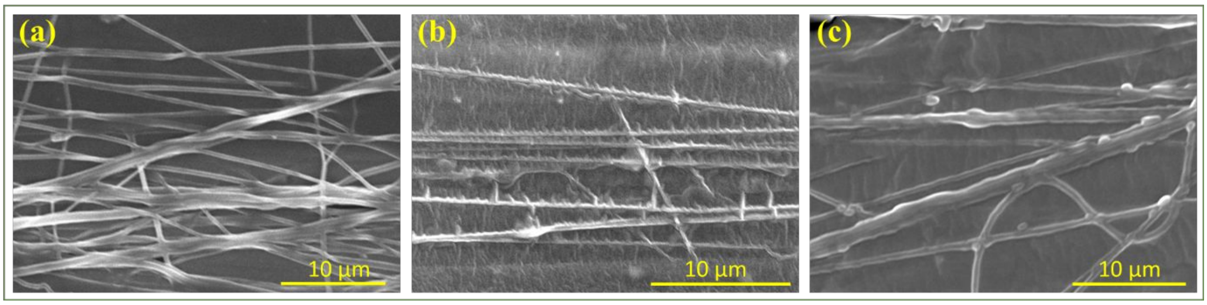

Figure S5. *SEM images of the untreated F7-Ag2 at different applied voltages during electrospinning (a) 17 kV, (b) 19 kV and (c) 21 kV.*

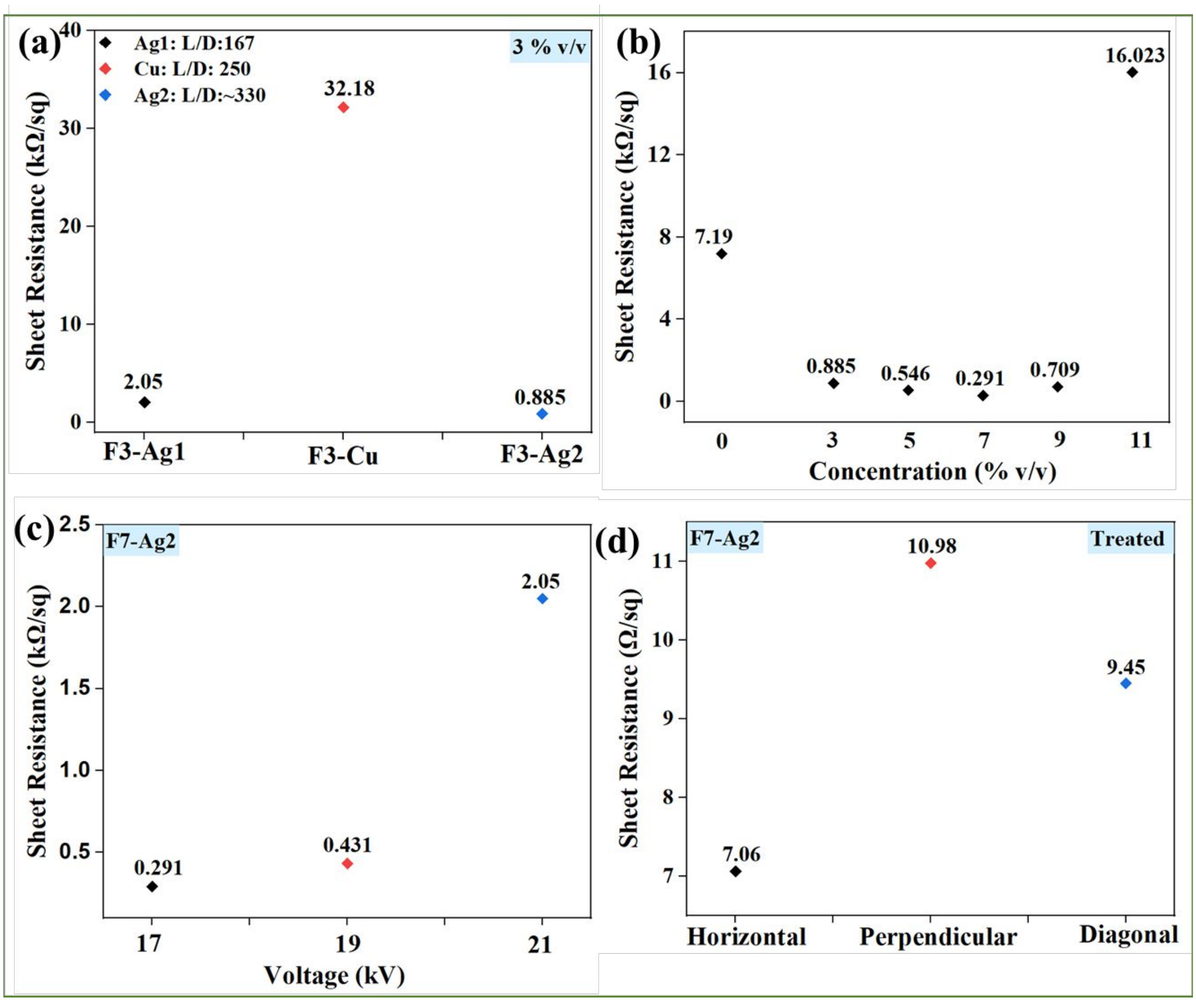

Figure S6. *Sheet resistance of untreated PEDOT:PSS fibres (a) F3-Ag1, F3-Cu and F3-Ag2, (b) with Ag2 in various concentrations, (c) Sheet resistance of untreated F7-Ag2 fibres with different applied voltages, (d) Sheet resistance of treated samples F7-Ag2 in different directions.*

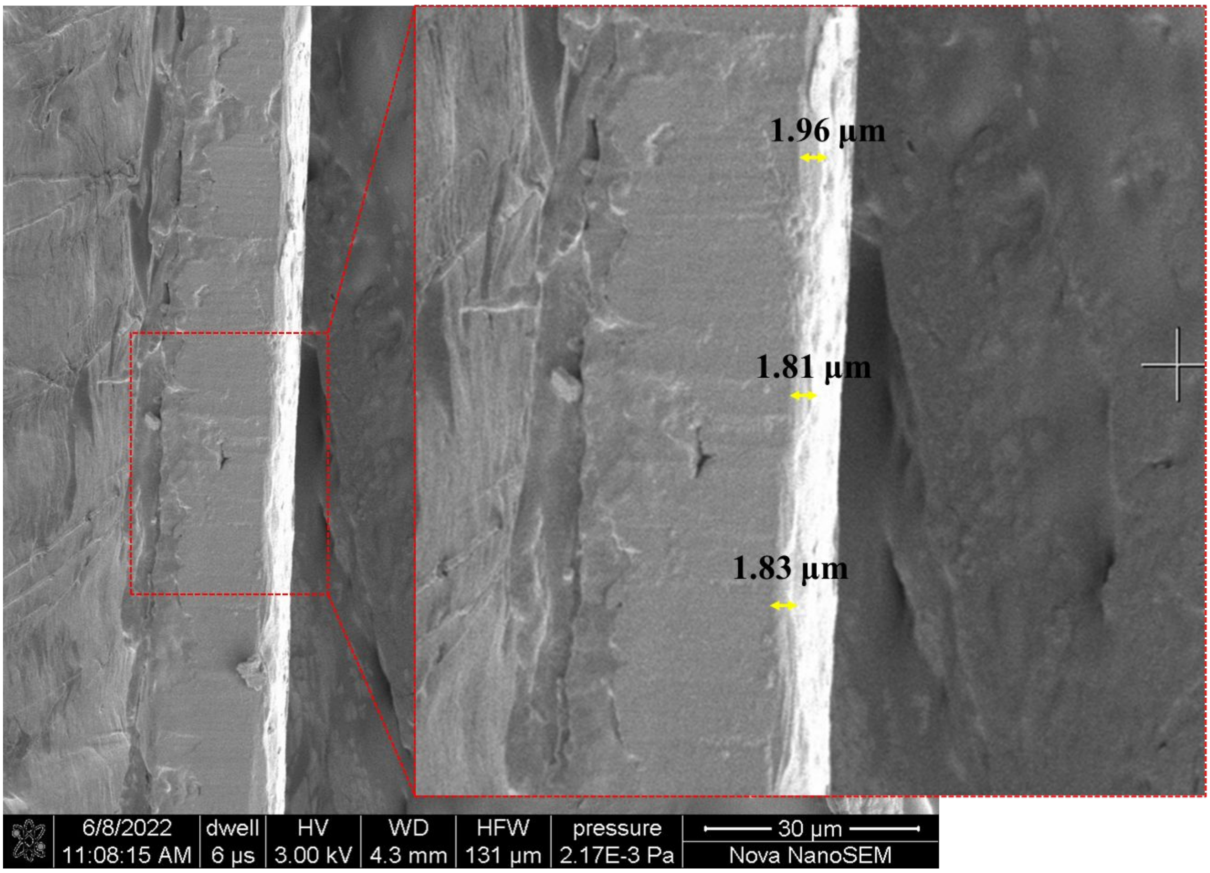

Figure S7. *SEM cross-section image of the treated F7-Ag2 fibres.*

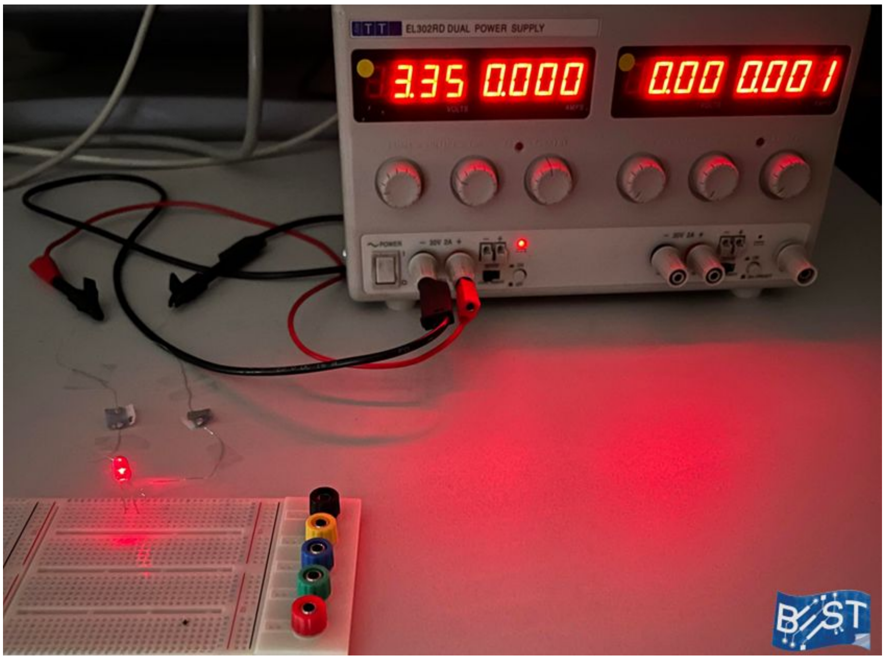

Figure S8. *LED circuit with F7-Ag2 electrospun for 30 mins.*

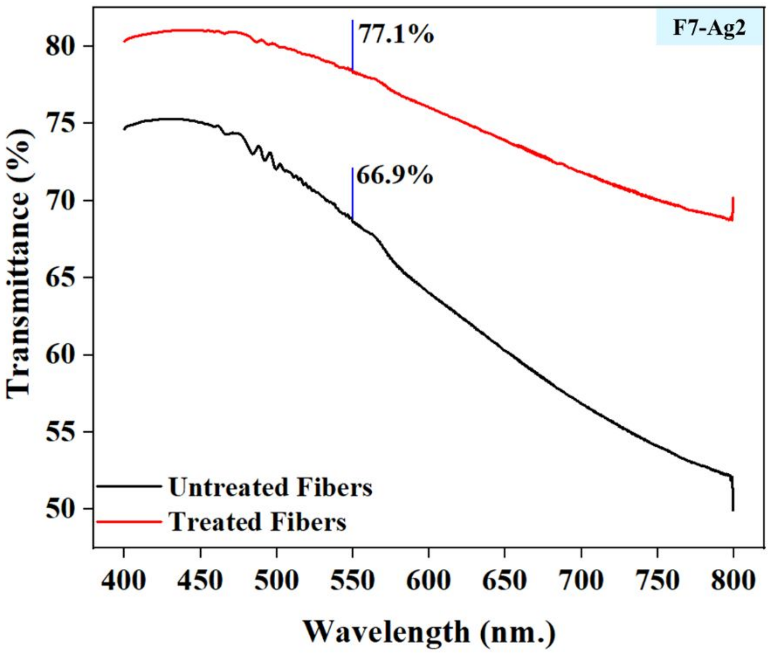

Figure S9. *Transmittance of untreated and treated sample F7-Ag2*

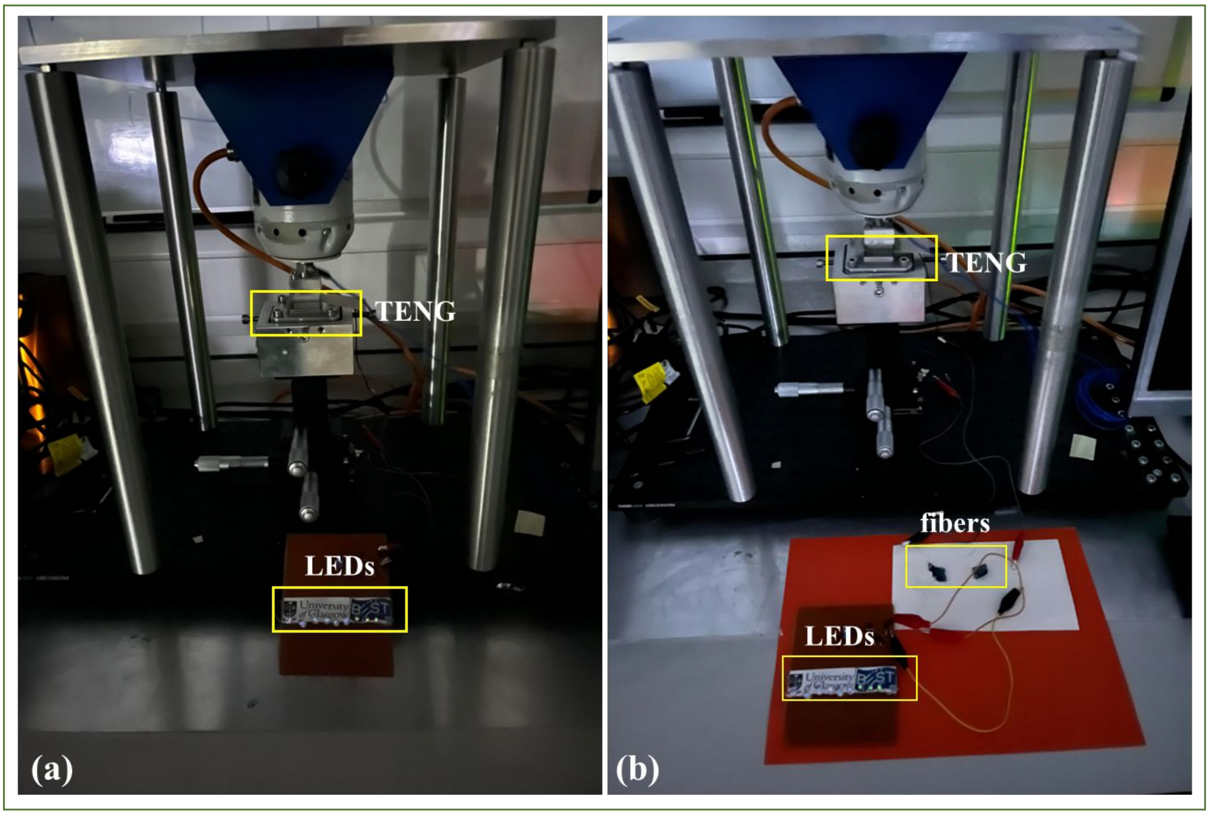

Figure S10. *(a) TENG with fibres as an electrode connected with an LED circuit and (b) TENG with fibres as an electrode and fibres as interconnects, connected with an LED circuit*

Table S5: Electrospinning parameters for the PEDOT:PSS/PEO/Nanowires solution

Sample	Nanowires	Voltage (kV)
**F0**	-	13
**F3-Ag1**	3 % v/v Ag[1*]	14
**F3-Cu**	3 % v/v Cu	15
**F3-Ag2**	3 % v/v Ag[2*]	15
**F5-Ag2**	5 % v/v Ag[2*]	16
**F7-Ag2**	7 % v/v Ag[2*]	17
**F9-Ag2**	9 % v/v Ag[2*]	18
**F11-Ag2**	11 % v/v Ag[2*]	19

[1*] refers to AgNWs with an aspect ratio (L/D): 167

[2*] refers to AgNWs with an aspect ratio (L/D): ~330

1. Abedi, A.; Hasanzadeh, M.; Tayebi, L., Conductive nanofibrous Chitosan/PEDOT:PSS tissue engineering scaffolds. *Materials Chemistry and Physics* **2019,** *237*, 121882.
2. Babaie, A.; Bakhshandeh, B.; Abedi, A.; Mohammadnejad, J.; Shabani, I.; Ardeshirylajimi, A.; Reza Moosavi, S.; Amini, J.; Tayebi, L., Synergistic effects of conductive PVA/PEDOT electrospun scaffolds and electrical stimulation for more effective neural tissue engineering. *European Polymer Journal* **2020,** *140*, 110051.
3. Chotimah, C.; Rianjanu, A.; Winardianto, B.; Munir, M.; Kartini, I.; Triyana, K., Electrospun Nanofiber Poly (3, 4-ethylenedioxytriophene): poly (styrene sulfonate)/poly (vinyl alcohol) as Strain Sensor Application. *Journal of Science and Applicative Technology* **2021,** *5* (2), 342-347.
4. Kara, M. O. P.; Frey, M. W., Effects of solvents on the morphology and conductivity of poly(3,4-ethylenedioxythiophene):Poly(styrenesulfonate) nanofibers. *Journal of Applied Polymer Science* **2014,** *131* (11).
5. Liu, N.; Fang, G.; Wan, J.; Zhou, H.; Long, H.; Zhao, X., Electrospun PEDOT:PSS–PVA nanofiber based ultrahigh-strain sensors with controllable electrical conductivity. *Journal of Materials Chemistry* **2011,** *21* (47), 18962-18966.
6. Sarabi, G. A.; Latifi, M.; Bagherzadeh, R., Align and random electrospun mat of PEDOT:PSS and PEDOT:PSS/RGO. *AIP Conference Proceedings* **2018,** *1920* (1), 020045.
7. Yin, J.; Bai, Y.; Lu, J.; Ma, J.; Zhang, Q.; Hong, W.; Jiao, T., Enhanced mechanical performances and high-conductivity of rGO/PEDOT:PSS/PVA composite fiber films via electrospinning strategy. *Colloids and Surfaces A: Physicochemical and Engineering Aspects* **2022,** *643*, 128791.
8. Liu, J.; Zhuo, Y.; Lu, J.; Wang, L.; Ren, X., Novel Conductive rGO/PEDOT:PSS/PVA Nanofibers by Electrospinning. *Integrated Ferroelectrics* **2022,** *229* (1), 305-312.
9. Jiang, X.; Ban, C.; Li, L.; Hao, J.; Shi, N.; Chen, W.; Gao, P., Electrospinning of BCNNTs/PVA/PEDOT composite nanofibers films for research thermoelectric performance. *Journal of Applied Polymer Science* **2022,** *139* (17), 52049.
10. Costa, E. L.; Muniz, E. C.; Cava, C. E., PEDOT:PSS/AgNWs nanofibers obtained by electrospun and the post-treatment via DMSO vapor exposure. *Synthetic Metals* **2023,** *298*, 117442.
11. Bessaire, B.; Mathieu, M.; Salles, V.; Yeghoyan, T.; Celle, C.; Simonato, J.-P.; Brioude, A., Synthesis of Continuous Conductive PEDOT:PSS Nanofibers by Electrospinning: A Conformal Coating for Optoelectronics. *ACS Applied Materials & Interfaces* **2017,** *9* (1), 950-957.
12. Cárdenas-Martínez, J.; España-Sánchez, B. L.; Esparza, R.; Ávila-Niño, J. A., Flexible and transparent supercapacitors using electrospun PEDOT:PSS electrodes. *Synthetic Metals* **2020,** *267*, 116436.
13. Latonen, R.-M.; Cabrera, J. A. W.; Lund, S.; Kosourov, S.; Vajravel, S.; Boeva, Z.; Wang, X.; Xu, C.; Allahverdiyeva, Y., Electrospinning of Electroconductive Water-Resistant

Nanofibers of PEDOT–PSS, Cellulose Nanofibrils and PEO: Fabrication, Characterization, and Cytocompatibility. *ACS Applied Bio Materials* **2021,** *4* (1), 483-493.

14. Lerond, M.; Skene, W. G.; Cicoira, F., Enhancing the performance of transparent and highly stretchable organic electrochemical transistors by acid treatment and copolymer blending of electrospun PEDOT: PSS fibers. *Journal of Materials Chemistry C* **2022**.

15. Zhao, W.; Yalcin, B.; Cakmak, M., Dynamic assembly of electrically conductive PEDOT:PSS nanofibers in electrospinning process studied by high speed video. *Synthetic Metals* **2015,** *203*, 107-116.

16. Massaglia, G.; Chiodoni, A.; Marasso, S. L.; Pirri, C. F.; Quaglio, M., Electrical Conductivity Modulation of Crosslinked Composite Nanofibers Based on PEO and PEDOT:PSS. *Journal of Nanomaterials* **2018,** *2018*, 3286901.

17. Choi, J.; Lee, J.; Choi, J.; Jung, D.; Shim, S. E., Electrospun PEDOT:PSS/PVP nanofibers as the chemiresistor in chemical vapour sensing. *Synthetic Metals* **2010,** *160* (13), 1415-1421.

18. Zhang, H.-D.; Yan, X.; Zhang, Z.-H.; Yu, G.-F.; Han, W.-P.; Zhang, J.-C.; Long, Y.-Z., Electrospun PEDOT:PSS/PVP Nanofibers for CO Gas Sensing with Quartz Crystal Microbalance Technique. *International Journal of Polymer Science* **2016,** *2016*, 3021353.

19. You, M.-H.; Wang, X.-X.; Yan, X.; Zhang, J.; Song, W.-Z.; Yu, M.; Fan, Z.-Y.; Ramakrishna, S.; Long, Y.-Z., A self-powered flexible hybrid piezoelectric–pyroelectric nanogenerator based on non-woven nanofiber membranes. *Journal of Materials Chemistry A* **2018,** *6* (8), 3500-3509.

20. Sun, B.; Long, Y.-Z.; Liu, S.-L.; Huang, Y.-Y.; Ma, J.; Zhang, H.-D.; Shen, G.; Xu, S., Fabrication of curled conducting polymer microfibrous arrays via a novel electrospinning method for stretchable strain sensors. *Nanoscale* **2013,** *5* (15), 7041-7045.

21. Huang, S.-R.; Lin, K.-F.; Don, T.-M.; Lee, C.-F.; Wang, M.-S.; Chiu, W.-Y., Thermoresponsive conductive polymer composite thin film and fiber mat: Crosslinked PEDOT:PSS and P(NIPAAm-co-NMA) composite. *Journal of Polymer Science Part A: Polymer Chemistry* **2016,** *54* (8), 1078-1087.

22. Lin, M.-F.; Don, T.-M.; Chang, F.-T.; Huang, S.-R.; Chiu, W.-Y., Preparation and properties of thermoresponsive and conductive composite fibers with core-sheath structure. *Journal of Polymer Science Part A: Polymer Chemistry* **2016,** *54* (9), 1299-1307.

23. Bhattacharya, S.; Kim, D.; Gopal, S.; Tice, A.; Lang, K.; Dordick, J. S.; Plawsky, J. L.; Linhardt, R. J., Antimicrobial effects of positively charged, conductive electrospun polymer fibers. *Materials Science and Engineering: C* **2020,** *116*, 111247.

24. Macagnano, A.; Zampetti, E.; Pantalei, S.; De Cesare, F.; Bearzotti, A.; Persaud, K. C., Nanofibrous PANI-based conductive polymers for trace gas analysis. *Thin Solid Films* **2011,** *520* (3), 978-985.

25. Im, J. S.; Kim, J. G.; Lee, S.-H.; Lee, Y.-S., Enhanced adhesion and dispersion of carbon nanotube in PANI/PEO electrospun fibers for shielding effectiveness of electromagnetic interference. *Colloids and Surfaces A: Physicochemical and Engineering Aspects* **2010,** *364* (1), 151-157.

26. Malakhova, Y. N.; Korovin, A. N.; Lapkin, D. A.; Malakhov, S. N.; Shcherban, V. V.; Pichkur, E. B.; Yakunin, S. N.; Demin, V. A.; Chvalun, S. N.; Erokhin, V., Planar and 3D fibrous polyaniline-based materials for memristive elements. *Soft Matter* **2017,** *13* (40), 7300-7306.

27. Spiers, M. E.; Nielsen, D. J.; Pavey, K. D.; Truong, Y. B.; Rutledge, G. C.; Kingshott, P.; Eldridge, D. S., Conductive, Acid-Doped Polyaniline Electrospun Nanofiber Gas Sensing Substrates Made Using a Facile Dissolution Method. *ACS Applied Materials & Interfaces* **2021,** *13* (44), 52950-52959.

28. Konuk Ege, G.; Yüce, H.; Akay, Ö.; Öner, H.; Genç, G., A fabrication and characterization of luffa/PANI/PEO biocomposite nanofibers by means of electrospinning. *Pigment & Resin Technology* **2021,** *ahead-of-print* (ahead-of-print).

29. Hou, X.; Zhou, Y.; Liu, Y.; Wang, L.; Wang, J., Coaxial electrospun flexible PANI//PU fibers as highly sensitive pH wearable sensor. *Journal of Materials Science* **2020,** *55* (33), 16033-16047.

30. Low, K.; Horner, C. B.; Li, C.; Ico, G.; Bosze, W.; Myung, N. V.; Nam, J., Composition-dependent sensing mechanism of electrospun conductive polymer composite nanofibers. *Sensors and Actuators B: Chemical* **2015,** *207*, 235-242.

31. Farkhondehnia, H.; Amani Tehran, M.; Zamani, F., Fabrication of Biocompatible PLGA/PCL/PANI Nanofibrous Scaffolds with Electrical Excitability. *Fibers and Polymers* **2018,** *19* (9), 1813-1819.

32. Savest, N.; Plamus, T.; Kütt, K.; Kallavus, U.; Viirsalu, M.; Tarasova, E.; Vassiljeva, V.; Krasnou, I.; Krumme, A., Electrospun conductive mats from PANi-ionic liquid blends. *Journal of Electrostatics* **2018,** *96*, 40-44.

33. Mahmoudifard, M.; Soleimani, M.; Hatamie, S.; Zamanlui, S.; Ranjbarvan, P.; Vossoughi, M.; Hosseinzadeh, S., The different fate of satellite cells on conductive composite electrospun nanofibers with graphene and graphene oxide nanosheets. *Biomedical Materials* **2016,** *11* (2), 025006.

34. Qavamnia, S. S.; Nasouri, K., Conductive polyacrylonitrile/polyaniline nanofibers prepared by electrospinning process. *Polymer Science Series A* **2015,** *57* (3), 343-349.

35. Wang, M.; Tremblay, P.-L.; Zhang, T., Optimizing the electrical conductivity of polyacrylonitrile/polyaniline with nickel nanoparticles for the enhanced electrostimulation of Schwann cells proliferation. *Bioelectrochemistry* **2021,** *140*, 107750.

36. Yu, Q.-Z.; Shi, M.-M.; Deng, M.; Wang, M.; Chen, H.-Z., Morphology and conductivity of polyaniline sub-micron fibers prepared by electrospinning. *Materials Science and Engineering: B* **2008,** *150* (1), 70-76.

37. Sarvi, A.; Chimello, V.; Silva, A. B.; Bretas, R. E. S.; Sundararaj, U., Coaxial electrospun nanofibers of poly(vinylidene fluoride)/polyaniline filled with multi-walled carbon nanotubes. *Polymer Composites* **2014,** *35* (6), 1198-1203.

38. McKeon, K. D.; Lewis, A.; Freeman, J. W., Electrospun poly(D,L-lactide) and polyaniline scaffold characterization. *Journal of Applied Polymer Science* **2010,** *115* (3), 1566-1572.

39. Xu, P.; Hussain, A. M.; Xu, X.; Cui, J.; Li, W.; Wang, G. In *Preparation and cytocompatibility of polyaniline/PLCL conductive nanofibers*, 2010 3rd International Conference on Biomedical Engineering and Informatics, 16-18 Oct. 2010; 2010; pp 1719-1722.

40. Massoumi, B.; Sarvari, R.; Agbolaghi, S., Biodegradable and conductive hyperbranched terpolymers based on aliphatic polyester, poly(D,L-lactide), and polyaniline used as scaffold in tissue engineering. *International Journal of Polymeric Materials and Polymeric Biomaterials* **2018,** *67* (13), 808-821.

41. Moutsatsou, P.; Coopman, K.; Georgiadou, S., Biocompatibility Assessment of Conducting PANI/Chitosan Nanofibers for Wound Healing Applications. *Polymers* **2017,** *9* (12).

42. Baştürk, E.; Çakmakçi, E.; Madakbaş, S.; Kahraman, M. V., Surface and proton conductivity properties of electrospun poly(vinyl butyral)/polyaniline nanofibers. *Advances in Polymer Technology* **2018,** *37* (6), 1774-1781.

43. Chen, C.-Y.; Huang, S. Y.; Wan, H.-Y.; Chen, Y.-T.; Yu, S.-K.; Wu, H.-C.; Yang, T.-I., Electrospun Hydrophobic Polyaniline/Silk Fibroin Electrochromic Nanofibers with Low Electrical Resistance. *Polymers* **2020,** *12* (9).

44. Massoumi, B.; Abbasian, M.; Jahanban-Esfahlan, R.; Mohammad-Rezaei, R.; Khalilzadeh, B.; Samadian, H.; Rezaei, A.; Derakhshankhah, H.; Jaymand, M., A novel bio-inspired conductive, biocompatible, and adhesive terpolymer based on polyaniline, polydopamine, and polylactide as scaffolding biomaterial for tissue engineering application. *International Journal of Biological Macromolecules* **2020,** *147*, 1174-1184.

45. Fotia, A.; Malara, A.; Paone, E.; Bonaccorsi, L.; Frontera, P.; Serrano, G.; Caneschi, A., Self Standing Mats of Blended Polyaniline Produced by Electrospinning. *Nanomaterials* **2021,** *11* (5).

46. Daraeinejad, Z.; Shabani, I., Enhancing biocompatibility of polyaniline-based scaffolds by using a bioactive dopant. *Synthetic Metals* **2021,** *271*, 116642.

47. Massoumi, B.; Aali, N.; Jaymand, M., Novel nanostructured star-shaped polyaniline derivatives and their electrospun nanofibers with gelatin. *RSC Advances* **2015,** *5* (130), 107680-107693.

48. Kai, D.; Prabhakaran, M. P.; Jin, G.; Ramakrishna, S., Polypyrrole-contained electrospun conductive nanofibrous membranes for cardiac tissue engineering. *Journal of Biomedical Materials Research Part A* **2011,** *99A* (3), 376-385.

49. Kang, T. S.; Lee, S. W.; Joo, J.; Lee, J. Y., Electrically conducting polypyrrole fibers spun by electrospinning. *Synthetic Metals* **2005,** *153* (1-3), 61-64.

50. Gois, B. H. S.; Bittencourt, J. C.; David-Parra, D. N.; Olivati, C. d. A.; Merlini, C.; Agostini, D. L. d. S., Electrospun PPY. DBSA/PVA Nanofibers for Ammonium Gas Sensor. *Materials Research* **2021,** *24*.
51. Maharjan, B.; Kaliannagounder, V. K.; Jang, S. R.; Awasthi, G. P.; Bhattarai, D. P.; Choukrani, G.; Park, C. H.; Kim, C. S., In-situ polymerized polypyrrole nanoparticles immobilized poly(ε-caprolactone) electrospun conductive scaffolds for bone tissue engineering. *Materials Science and Engineering: C* **2020,** *114*, 111056.
52. Tavakkol, E.; Tavanai, H.; Abdolmaleki, A.; Morshed, M., Production of conductive electrospun polypyrrole/poly(vinyl pyrrolidone) nanofibers. *Synthetic Metals* **2017,** *231*, 95-106.
53. Zarei, M.; Samimi, A.; Khorram, M.; Abdi, M. M.; Golestaneh, S. I., Fabrication and characterization of conductive polypyrrole/chitosan/collagen electrospun nanofiber scaffold for tissue engineering application. *International Journal of Biological Macromolecules* **2021,** *168*, 175-186.
54. Cong, Y.; Liu, S.; Chen, H., Fabrication of Conductive Polypyrrole Nanofibers by Electrospinning. *Journal of Nanomaterials* **2013,** *2013*, 148347.
55. de Castro, J. G.; Rodrigues, B. V. M.; Ricci, R.; Costa, M. M.; Ribeiro, A. F. C.; Marciano, F. R.; Lobo, A. O., Designing a novel nanocomposite for bone tissue engineering using electrospun conductive PBAT/polypyrrole as a scaffold to direct nanohydroxyapatite electrodeposition. *RSC Advances* **2016,** *6* (39), 32615-32623.
56. Zhou, Z.-F.; Zhang, F.; Wang, J.-G.; Chen, Q.-C.; Yang, W.-Z.; He, N.; Jiang, Y.-Y.; Chen, F.; Liu, J.-J., Electrospinning of PELA/PPY Fibrous Conduits: Promoting Peripheral Nerve Regeneration in Rats by Self-Originated Electrical Stimulation. *ACS Biomaterials Science & Engineering* **2016,** *2* (9), 1572-1581.
57. Massoumi, B.; Jaymand, M., Chemical and electrochemical grafting of polythiophene onto poly(methyl methacrylate), and its electrospun nanofibers with gelatin. *Journal of Materials Science: Materials in Electronics* **2016,** *27* (12), 12803-12812.
58. Massoumi, B.; Massoumi, R.; Aali, N.; Jaymand, M., Novel nanostructured star-shaped polythiophene, and its electrospun nanofibers with gelatin. *Journal of Polymer Research* **2016,** *23* (7), 136.
59. Park, J.; Kaliannagounder, V. K.; Jang, S. R.; Yoon, D.; Rezk, A. I.; Bhattarai, D. P.; Kim, C. S., Electroconductive Polythiophene Nanocomposite Fibrous Scaffolds for Enhanced Osteogenic Differentiation via Electrical Stimulation. *ACS Biomaterials Science & Engineering* **2022,** *8* (5), 1975-1986.
60. Hatamzadeh, M.; Najafi-Moghadam, P.; Baradar-Khoshfetrat, A.; Jaymand, M.; Massoumi, B., Novel nanofibrous electrically conductive scaffolds based on poly(ethylene glycol)s-modified polythiophene and poly(ε-caprolactone) for tissue engineering applications. *Polymer* **2016,** *107*, 177-190.

61. Hatamzadeh, M.; Najafi-Moghadam, P.; Beygi-Khosrowshahi, Y.; Massoumi, B.; Jaymand, M., Electrically conductive nanofibrous scaffolds based on poly(ethylene glycol)s-modified polyaniline and poly(ε-caprolactone) for tissue engineering applications. *RSC Advances* **2016,** *6* (107), 105371-105386.

62. Jaymand, M.; Sarvari, R.; Abbaszadeh, P.; Massoumi, B.; Eskandani, M.; Beygi-Khosrowshahi, Y., Development of novel electrically conductive scaffold based on hyperbranched polyester and polythiophene for tissue engineering applications. *Journal of Biomedical Materials Research Part A* **2016,** *104* (11), 2673-2684.

63. Sanfelice, R. C.; Mercante, L. A.; Pavinatto, A.; Tomazio, N. B.; Mendonça, C. R.; Ribeiro, S. J. L.; Mattoso, L. H. C.; Correa, D. S., Hybrid composite material based on polythiophene derivative nanofibers modified with gold nanoparticles for optoelectronics applications. *Journal of Materials Science* **2017,** *52* (4), 1919-1929.

64. Sarvari, R.; Massoumi, B.; Zareh, A.; Beygi-Khosrowshahi, Y.; Agbolaghi, S., Porous conductive and biocompatible scaffolds on the basis of polycaprolactone and polythiophene for scaffolding. *Polymer Bulletin* **2020,** *77* (4), 1829-1846.
